# Genetic and Biochemical Dissection of a HisKA Domain Identifies Residues Required Exclusively for Kinase and Phosphatase Activities

**DOI:** 10.1371/journal.pgen.1003084

**Published:** 2012-11-29

**Authors:** Jonathan W. Willett, John R. Kirby

**Affiliations:** Department of Microbiology, University of Iowa, Iowa City, Iowa, United States of America; University of Geneva Medical School, Switzerland

## Abstract

Two-component signal transduction systems, composed of histidine kinases (HK) and response regulators (RR), allow bacteria to respond to diverse environmental stimuli. The HK can control both phosphorylation and subsequent dephosphorylation of its cognate RR. The majority of HKs utilize the HisKA subfamily of dimerization and histidine phosphotransfer (DHp) domains, which contain the phospho-accepting histidine and directly contact the RR. Extensive genetics, biochemistry, and structural biology on several prototypical TCS systems including NtrB-NtrC and EnvZ-OmpR have provided a solid basis for understanding the function of HK–RR signaling. Recently, work on NarX, a HisKA_3 subfamily protein, indicated that two residues in the highly conserved region of the DHp domain are responsible for phosphatase activity. In this study we have carried out both genetic and biochemical analyses on *Myxococcus xanthus* CrdS, a member of the HisKA subfamily of bacterial HKs. CrdS is required for the regulation of spore formation in response to environmental stress. Following alanine-scanning mutagenesis of the α1 helix of the DHp domain of CrdS, we determined the role for each mutant protein for both kinase and phosphatase activity. Our results indicate that the conserved acidic residue (E372) immediately adjacent to the site of autophosphorylation (H371) is specifically required for kinase activity but not for phosphatase activity. Conversely, we found that the conserved Thr/Asn residue (N375) was required for phosphatase activity but not for kinase activity. We extended our biochemical analyses to two CrdS homologs from *M. xanthus*, HK1190 and HK4262, as well as *Thermotoga maritima* HK853. The results were similar for each HisKA family protein where the conserved acidic residue is required for kinase activity while the conserved Thr/Asn residue is required for phosphatase activity. These data are consistent with conserved mechanisms for kinase and phosphatase activities in the broadly occurring HisKA family of sensor kinases in bacteria.

## Author Summary

Bacterial histidine kinases (HK) serve as bifunctional enzymes capable of both phosphorylation and dephosphorylation of their cognate response regulators (RR). The majority of HKs (77%) belong to the HisKA subfamily. While both kinase and phosphatase functions have been assayed for HisKA proteins, relatively few examples have been studied to determine which residues are required for kinase and phosphatase activity. Recent studies on NarX, a HisKA_3 family protein, and the dedicated phosphatases CheZ and CheX illustrate requirements for two amino acids for phosphatase function. In this study, we undertook saturating mutagenesis of the proposed interaction surface between the HK and its cognate RR and conclude that only one residue (T/N) is required exclusively for phosphatase activity for HisKA family proteins in evolutionarily distant organisms *Myxococcus xanthus* and *Thermotoga maritima*. In addition, we identified only one residue (E/D), adjacent to the conserved site of phosphorylation, required exclusively for kinase activity within the highly conserved motif H-E/D-x-x-T/N. Because similar sequences are found in nearly all HisKA kinases, these residues provide excellent targets for dissection of kinase and phosphatase activities within this broadly occurring family of bacterial kinases.

## Introduction

**Figure 1:**
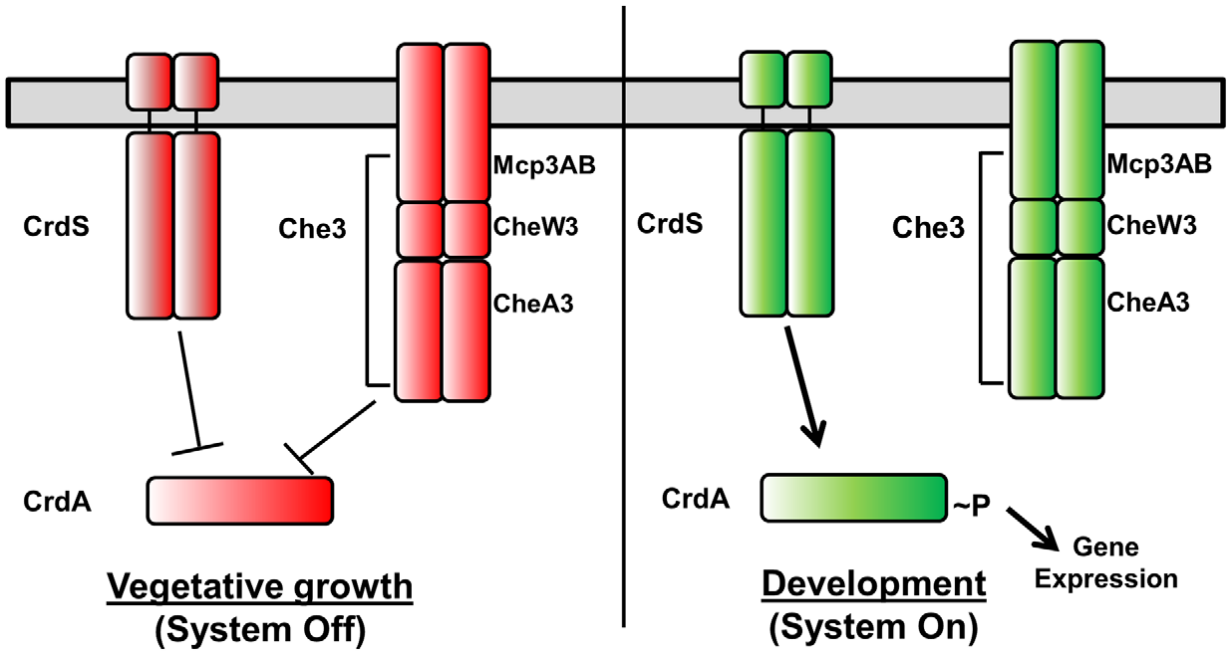
Model for Cross Regulation of CrdA by CrdS and the Che3 Chemosensory System. In absence of appropriate developmental signals (e.g. starvation), both the Che3 system and CrdS act as phosphatases to maintain CrdA in an unphosphorylated and inactive state. Following stimulation by nutrient deprivation, CrdS kinase activity is increased, resulting in phosphorylation of CrdA thereby regulating gene expression required for proper timing of developmental aggregation and sporulation.

*Myxococcus xanthus* serves as a model for prokaryotic development. Upon sensing nutrient limitation, *M. xanthus* undergoes a complex signal transduction cascade culminating in the formation of macroscopic fruiting bodies composed of metabolically dormant myxospores. Entry into development is regulated such that *M. xanthus* is able to coordinate cell motility and sporulation in response to a diverse set of environmental cues. Two-component systems (TCS) directly link environmental signals to changes in motility and gene expression required for *M. xanthus* developmental processes [1]. In order to respond to a variety of environmental stimuli, *M. xanthus* encodes a large number of TCS proteins (122 prototypical histidine kinases (HK) and 127 response regulators (RR)) [2]. One important developmental regulator, CrdA, is a σ^54^-dependent transcription factor homolog of NtrC which regulates the timing of aggregation required for fruiting body development [3]. CrdA activity is regulated via phosphorylation and dephosphorylation by the histidine kinase CrdS and the Che3 Chemosensory System. Our previous work demonstrated that CrdS is a bifunctional enzyme capable of both kinase and phosphatase activities which directly control CrdA activity. CheA3 within the Che3 pathway synergistically acts with CrdS to regulate CrdA∼P levels within the cell (Figure 1) [4].

The prototypical TCS provides a mechanism for bacteria to sense and respond to extracellular changes in stimuli. A diverse set of cellular processes such as virulence, stress responses, metabolism, motility and bacterial development are regulated by TCS. TCS are comprised of two proteins, the HK and the RR. Signal transduction begins when HK activity is stimulated by binding to the appropriate ligand, usually via an N-terminal membrane bound extracellular sensing domain. HKs are multidomain proteins displaying wide variation in domain organization and composition but are minimally characterized by the presence of a dimerization and histidine phosphotransfer (DHp) domain and a catalytic and ATP-binding domain (CA) [5]. After ATP is bound by the CA domain, the γ-phosphoryl group is transferred to the conserved His residue within the DHp domain [6]. The HK phosphoryl group is then able to undergo phosphotransfer to a conserved Asp residue within the receiver (REC) domain of the RR. The phosphorylated version of the protein (RR∼P) mediates the appropriate cellular response.

In order to maintain proper signaling fidelity, the ratio of RR to RR∼P must be carefully monitored. Although phosphorylated RRs can undergo autodephosphorylation, it is common for this activity to be augmented by additional proteins. In the case of bacterial chemotaxis or the *Bacillus subtilis* sporulation pathway, discrete phosphatases are utilized such as CheX, CheZ or RapH [7]–[9]. In addition to or in lieu of discrete phosphatases, many HKs possess inherent phosphatase activity for their cognate RR∼P [4], [10]–[12]. Phosphatase activity is important for limiting cross-talk from non-cognate kinases or from high-energy, small-molecule donors such as acetyl-phosphate (AcP), and serves to enhance turnover of the phosphorylated RR following loss of signal [13]. By regulating both phosphatase and kinase activities of the HK, cells can carefully tune the level of phosphorylated RR.

The mechanism for RR∼P dephosphorylation requires the correct positioning of a nucleophilic water molecule to hydrolyze the phospho-aspartate bond within the receiver domain [14]. The auxiliary phosphatases CheX and CheZ require both an acidic amino acid and an amide (ExxN and DxxxQ, respectively) to coordinate a water molecule for nucleophilic attack on phospho-CheY [7]. A similar motif has been found for NarX where the DxxxQ sequence is required for phosphatase activity. The DxxxQ motif appears to be highly conserved in HKs containing the HisKA_3 subfamily of DHp domains, suggesting a general mechanism for phosphatase activity in this subfamily [12]. Based on these findings, it was proposed that HisKA domains may have a similar two-residue requirement within the E/DxxT/N motif for phosphatase activity [13]. In this study we tested that hypothesis and demonstrate that only one amino acid, the highly conserved T/N residue, is required for phosphatase activity in the tested HisKA family proteins. Thus HisKA and HisKA_3 proteins appear to utilize different mechanisms for phosphatase activity. Additionally we demonstrate that the conserved E/D residue is specifically required for kinase activity.

There are five HK subfamilies designated by amino acid composition and position within the DHp domains. The HisKA subfamily is the most abundant comprising 77% of DHp domains found within 1500 sequenced microbial genomes [2], [15]. Included within this subfamily are the well-studied histidine kinases, NtrB, PhoQ and EnvZ of *E. coli*, as well as CrdS from *M. xanthus*. For NtrB and EnvZ of *E. coli*, the DHp domain is sufficient for phosphatase activity, although the presence of the CA domain greatly increases activity [16]. *In vivo* expression of the DHp domain of either NtrB or EnvZ is sufficient to generate a constitutive phosphatase phenotype. *In vitro* work further demonstrated that the DHp domains of NtrB and EnvZ are required to dephosphorylate the cognate RRs, NtrC and OmpR, respectively [16], [17]. Mutational analyses of EnvZ and NtrB identified additional residues within the DHp domains that altered kinase or phosphatase activities [10], [18]–[22].

**Figure 2:**
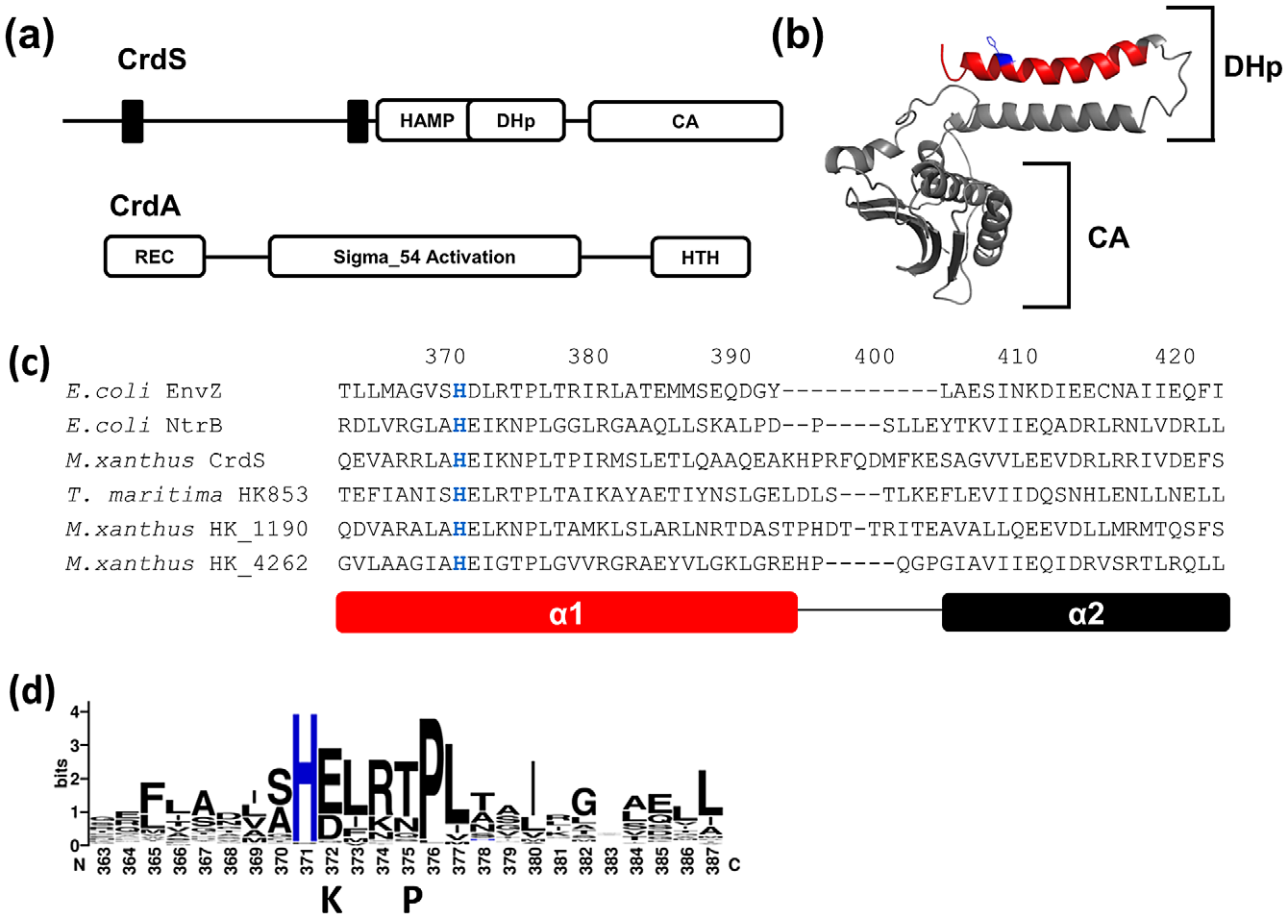
Sequence Analysis of CrdS and HisKA Domains. (A) Domain organization of the histidine kinase, CrdS, and its cognate response regulator, CrdA. CrdS is composed of a predicted N-terminal periplasmic sensing domain followed by HAMP, DHp and CA domains. CrdA is a σ54-dependent transcription factor regulating developmental gene expression. (B) Predicted structure of the soluble portion of CrdS after threading onto the structure of *T. maritima* HK853. The DHp domain (spanning α1 and α2 helices) and CA domain are indicated by brackets. The α1 helix of CrdS (red) containing the conserved phosphorylatable histidine (blue) was subjected to mutagenesis. (C) Sequence alignment of CrdS with other HisKA DHp domains spanning the α1 (red) and α2 helices. Residues are numbered relative to their position in CrdS. (D) Sequence conservation across α1 helices of the 100 most diverse HisKA DHp domains. “K” and “P” indicate residues required exclusively for kinase and phosphatase activity, respectively.

Recently, a co-structure of the *Thermotoga maritima* TCS, HK853-RR468, demonstrated that α1 of the DHp domain directly contacts the RR such that residues within α1 are positioned to coordinate a water molecule for nucleophilic attack of the phospho-aspartate within the RR [23]. Additional work has demonstrated that residues responsible for the specific interaction between the HK and RR are located within the α1 helix of the DHp domain [24], [25]. Because the DHp α1 helix is the primary site of interaction between the HK and RR and the DHp domain is sufficient for phosphatase activity, the results suggest that residues within the DHp domain (Figure 2) are solely responsible for coordination of the nucleophilic water required for RR∼P dephosphorylation.

In this study, we have identified residues within the DHp α1 helix of the HisKA domain of CrdS that are required exclusively for kinase or phosphatase activity as well as several residues that tolerate substitutions. Each residue within CrdS DHp α1 helix was mutated using alanine-scanning mutagenesis and tested using a series of *in vitro* biochemical assays. Our data indicate that substitution of only one residue, N375, eliminates phosphatase activity without significantly affecting kinase activity for CrdS. Conversely, the acidic residue adjacent to the conserved histidine, E372, is required for kinase activity but is not required for phosphatase activity. *In vivo* expression of the kinase or phosphatase deficient proteins in *M. xanthus* resulted in predicted phenotypes: a kinase dead (K−) mutant is delayed for development and a phosphatase dead (P−) mutant is premature for development. We subsequently extended our analyses to other histidine kinases by making similar mutations within *T. maritima* HK853 and *M. xanthus* NtrB homologs, HK4262 and HK1190. In total, our data indicate that conserved residues most likely perform similar functions in homologous HisKA signaling systems. Overall, our results allow us to refine the model for HisKA subfamily function such that only one residue, corresponding to CrdS-N375, is required exclusively for phosphatase activity. This is distinct from the requirement observed for the HisKA_3 protein, NarX, as well as for CheC, CheZ and CheX phosphatases, each of which utilize two residues for phosphatase activity.

## Results

### Identification of Conserved Residues within CrdS and Other *M. xanthus* HisKA Family Proteins

Encoded within the genome of *M. xanthus* are 122 prototypical histidine kinases (HK) comprised of a DHp and CA domain. 118 of these HKs contain the HisKA subfamily DHp domain, including CrdS. Of the remaining four kinases, two possess the HisKA_3 subfamily DHp domain and two possess the His_kinase subfamily DHp domain [2]. The domain organization for CrdS, a prototypical HK, and its target response regulator, CrdA, are shown for comparison (Figure 2A). The majority of HKs in Bacteria belong to the HisKA subfamily and include NtrB and EnvZ of *E. coli* and HK853 of *T. maritima*. Because crystallographic analysis of multiple HKs have revealed similar structures, we threaded CrdS onto the structure of HK853 from *T. maritima* (Figure 2B) [26]. HisKA family DHp domains are composed of two antiparallel alpha helices termed α1 and α2, with the conserved phosphorylatable histidine located in the α1 helix (Figure 2B–2C). Two DHp subunits will dimerize thereby forming a four helix bundle [27]. The attached CA domain is composed of 5 β sheets and 3 short α helices (Figure 2B).

Next we performed sequence alignments of the region spanning α1 and α2 using *E. coli* NtrB and EnvZ, *T. maritima* HK853 and *M. xanthus* CrdS, HK1190 and HK4262 (Figure 2C). Even though *M. xanthus* and *E. coli* are distantly related proteobacteria, both NtrB and CrdS share an identical 9 amino acid sequence spanning the conserved histidine [L-A-H-E-I-K-N-P-L] indicating a likely role for these residues in either kinase or phosphatase function [28]. Interestingly, the *M. xanthus* HKs, CrdS, HK1190 and HK4262 have additional amino acids near the α1-α2 linker region compared to EnvZ and NtrB indicating that the *M. xanthus* proteins have either a larger linker and/or slightly longer α1 and α2 helices within the HisKA domain.

To identify residues that are highly conserved in *M. xanthus* HisKA domains, we generated a Weblogo plot based on a sequence alignment of the 100 most diverse HisKA domains (Figure 2D) found in sequenced bacterial genomes [29]. This Weblogo is very similar to that previously generated for HisKA domains, although that study used fewer representative sequences from two organisms, *E. coli* and *B. subtilis* [12]. As can be seen in Figure 2D, the phosphorylatable histidine is conserved in 100% of the sequences while the adjacent 6 amino acids display high sequence conservation. The conserved sequence, H-E/D-L-R-T/N-P-L, is similar to the H-box consensus sequence in the HPK1 family previously identified by Grebe *et al* [28]. For comparison to this diverse data set, we also generated the corresponding Weblogo for all 118 *M. xanthus* HisKA domains (Figure S1) [30]. The consensus sequence for the *M. xanthus* HisKA domains is very similar to that seen in the 100 most diverse HisKA domains, where both display high conservation over the six residues adjacent to the site of phosphorylation. The observed sequence similarity indicates a common requirement for these residues within kinases containing the HisKA subfamily of DHp domains.

### Mutagenesis of CrdS Identifies Residues Required Exclusively for Kinase Activity

While histidine kinases are highly conserved signaling molecules found throughout bacteria and have been widely investigated for biological function, a role for each of the highly conserved residues within DHp domains are not yet fully understood. This was recently demonstrated by the study on a HisKA_3 subfamily kinase, NarX from *E. coli*, where Huynh *et al.* identified two conserved residues required for phosphatase activity [12]. HisKA_3 domains are distinct from other DHp domains both structurally and with regard to conserved residues surrounding the phosphorylatable histidine, suggesting that different residues are required for activity within each subfamily of HK [13].

Arguably the best studied HK in the HisKA subfamily is EnvZ from *E. coli*. Mutagenesis of *envZ* has been performed using both cysteine- and alanine-scanning mutagenesis. However, those studies were not saturating and instead focused on either surface exposed residues or random cysteine mutagenesis [21], [22]. Additionally, no systematic mutagenesis has been performed on any *M. xanthus* HK, including the previously characterized HK CrdS. In order to define a role for each residue on HK activity, we generated a total of 38 mutants in DHp α-helix 1 of the HisKA domain of CrdS. 25 of the 38 total mutants were generated using alanine-scanning mutagenesis which should not disrupt the helical structure of the protein. Two of the residues in the α1 helix are alanine and were mutated to serine, a small non-charged amino acid that is unlikely to affect secondary structure. We made additional substitutions within the highly conserved region directly adjacent to the conserved H371. For purification of each protein, we used a construct which expresses a soluble portion of CrdS (amino acids 346–578) with the membrane spanning domain replaced by a 6x-His affinity tag [4]. For each mutant, we were able to express and purify a stable and soluble protein as indicated by gel-electrophoresis (Figure S2). Importantly, each of the key mutants affecting function maintain WT secondary structure as determined by circular dichroism (Figure S3).

**Figure 3:**
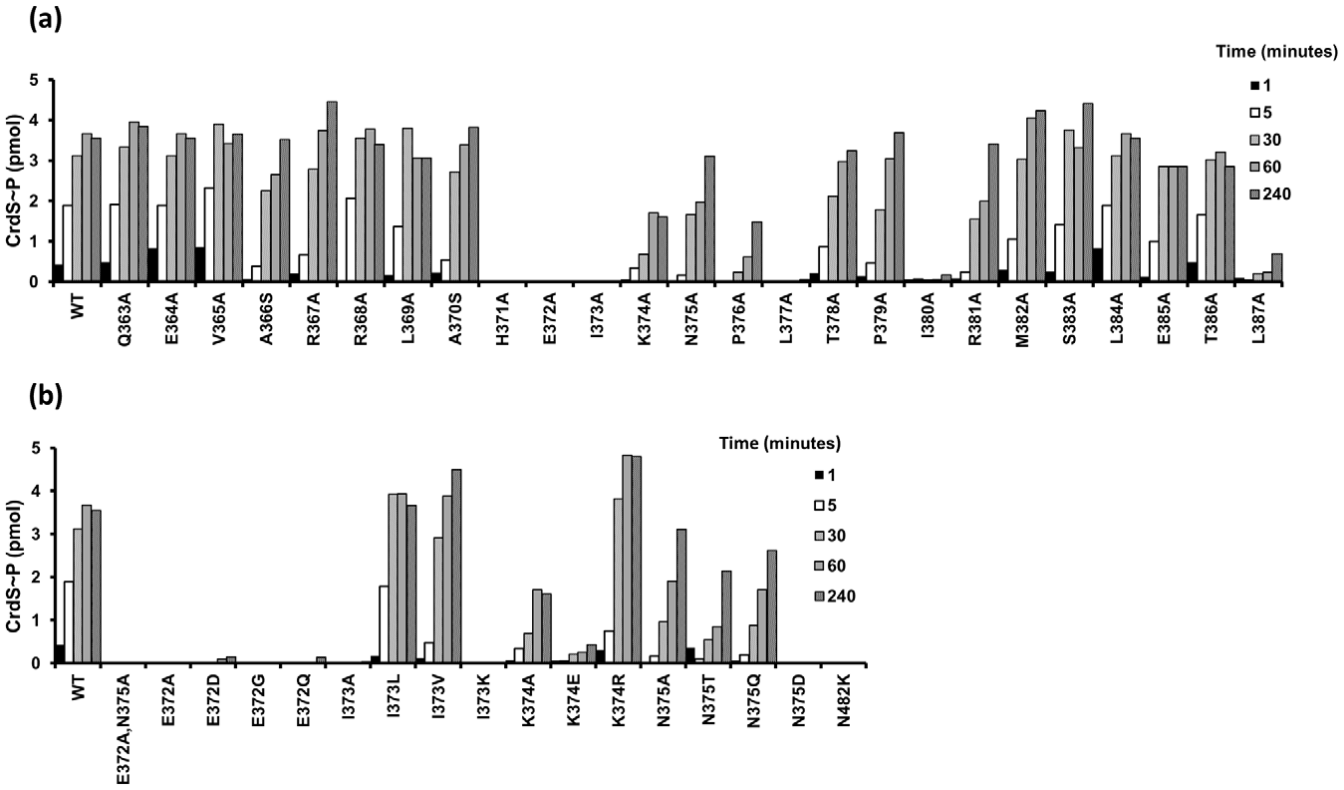
Kinase Activity of CrdS α1 Mutants. Purified WT and mutant proteins were diluted to 5 µM and allowed to autophosphorylate for 1, 5, 30, 60 and 240 minutes before the reactions were terminated and quantified (pmol of phosphorylated CrdS proteins shown on the y-axis). Amino acid substitutions were generated by site-directed mutagenesis and are indicated along the x-axis with numbers corresponding to the amino acid position of CrdS. Data shown is that of a representative set. (A) Autophosphorylation levels are shown for each alanine (or serine) substitution mutant protein relative to CrdS-WT shown at left. (B) Autophosphorylation of additional substitution mutants generated within the highly conserved region of CrdS. WT CrdS is shown at left.

To analyze the affect each substitution has on CrdS kinase activity, we performed *in vitro* autophosphorylation assays to measure the rate and amount of CrdS∼P generated. Each reaction containing 5 µM CrdS (wild type or mutant) was incubated with excess ATP (containing [γ-^32^P]-ATP) and allowed to proceed for 1, 5, 30, 60 or 240 minutes. Reactions were stopped by addition of SDS-loading buffer and samples were visualized after SDS-PAGE and subsequent exposure to phosphor screens for quantification. Our previous work demonstrated that WT CrdS reaches maximal phosphorylation by 60 minutes and is shown as a control (Figure 3A) [4]. Of the alanine mutants generated, three lacked any detectable kinase activity (K−) at four hours: H371A, E372A, and I373A. As expected, the substitution in the phosphorylatable His, H371A, eliminated kinase activity as shown previously [4]. Interestingly, all three substitution mutants completely lacking kinase activity are found within the highly conserved region (Figure 2) and span three consecutive residues, H371, E372 and I373 (Figure 3A). Additionally, the L377A and I380A mutant proteins were severely affected, reaching phosphorylation levels less than 1% of wild-type by four hours.

The E372A substitution adjacent to the phosphorylatable histidine, H371, lacks measurable kinase activity while retaining WT secondary structure (Figure 3A, Figure S3). Because the sequence conservation depicted in Figure 2D indicates that glutamate and aspartate occur at relatively equal frequencies, we also constructed the E372D mutation. Surprisingly, the CrdS-E372D mutant did not display any detectable kinase activity within four hours, despite the conservative substitution (Figure 3B). Additionally, the E372Q and E372G mutants also lack kinase activity, indicating that CrdS cannot tolerate substitutions at E372, suggesting a critical role for the position of the acidic side chain relative to the histidine for kinase activity.

Similar to the E372A mutation, the adjacent I373A substitution also abolished CrdS kinase activity. Conservation within HisKA proteins (Figure 2D) indicates that either leucine or valine could substitute for isoleucine at this position. Indeed, both the I373V or I373L substitutions did not disrupt kinase function (Figure 3B). To directly assay a requirement for hydrophobicity at this position, we also assayed the I373K mutant protein which was found to completely block kinase activity. Thus, it appears there is flexibility at position I373 of CrdS as long as the residue is hydrophobic.

Further mutagenesis of CrdS demonstrated the importance of two other residues for kinase activity, K374 and P376. While a K374E substitution dramatically reduced kinase activity, the conservative K374R substitution restores kinase activity to better than WT levels. Thus, CrdS requires a basic residue at position 374 for full kinase activity. Additionally, a P376A substitution resulted in decreased kinase activity and was able to reach ∼30–50% that of WT phosphorylation, similar to the K374A mutant protein.

In contrast, the N375A substitution within the highly conserved region had little effect on kinase function, displaying the highest level of kinase activity of all the mutants tested (∼87% of WT phosphorylation at 4 hours). While the rate of autophosphorylation for the N375A mutant is altered slightly, this does not appear to be significant since expression of *crdS-N375A* can complement a *ΔcrdS* mutant *in vivo* (discussed below; Figure 6, Figure S4). Sequence conservation indicates that N375 occurs at a frequency nearly equal to that of threonine (Figure 2D). To test flexibility at this position, we generated the N375T substitution mutant yielding a protein that retained approximately 60% of WT kinase activity (at 4 hours). Another substitution, N375Q, also resulted in a ∼40% reduction in kinase activity while the N375D substitution exhibited no measurable kinase activity (Figure 3B). Lastly, as a control for our kinase assays, a CrdS N482K mutant was generated at a position previously demonstrated to be required for CA domain function in the HKs, CckA and EnvZ [19], [31]. As expected, the CrdS-N482K mutant resulted in a kinase deficient protein, indicating the requirement of the CA domain for kinase activity (Figure 3B).

### Residue N375 of CrdS Is Required Exclusively for Phosphatase Activity

**Figure 4:**
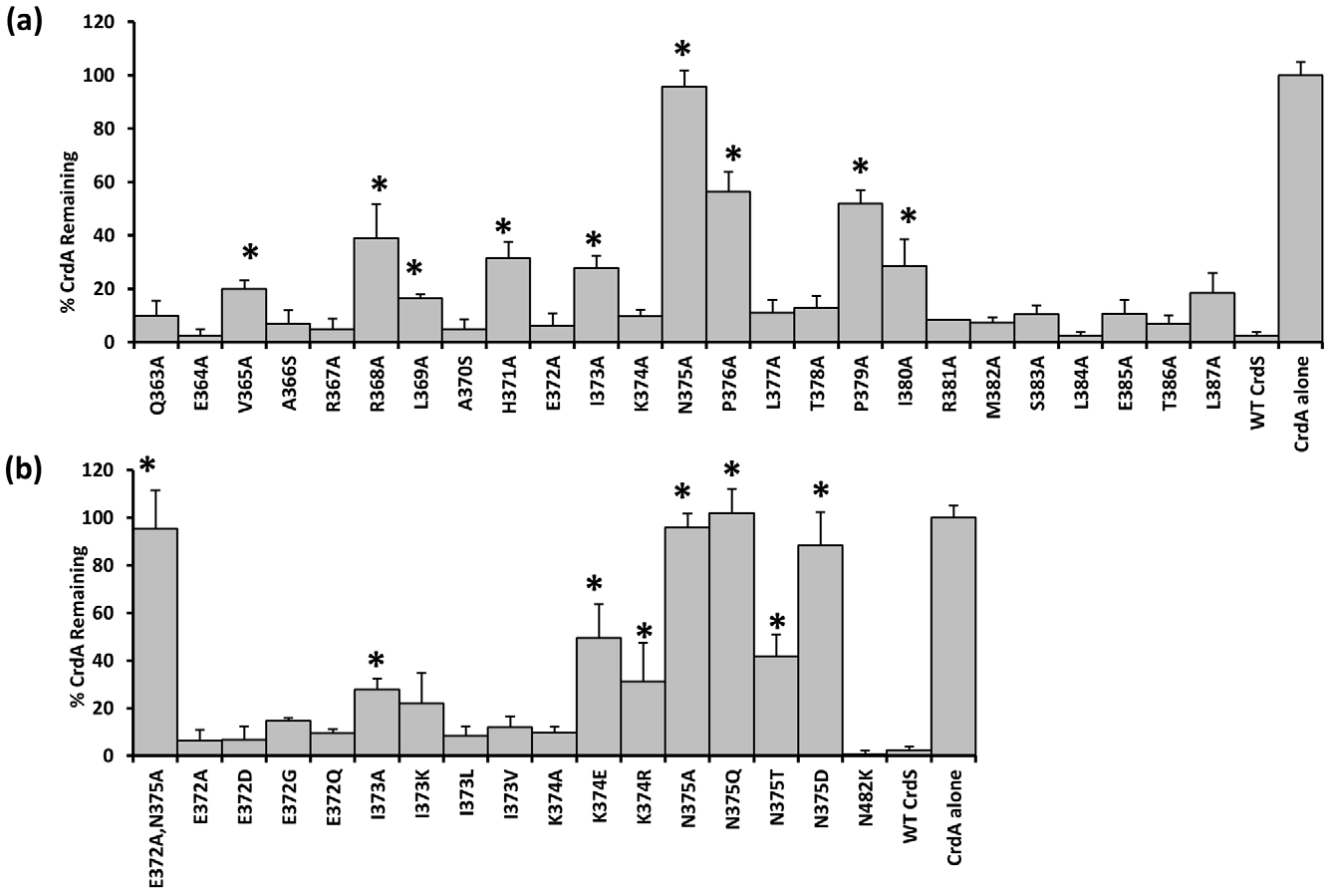
Phosphatase Activity of CrdS α1 Mutants. CrdS WT and mutant proteins were tested for their ability to dephosphorylate CrdA∼P which was generated using [^32^P] acetyl-phosphate. Bars indicate the amount of CrdA∼P remaining after a 5 minute incubation with each CrdS mutant protein. Data shown are the average of three separate experiments with error bars indicating standard deviation. Statistically significant differences in phosphatase activity are indicated by an (*), p<0.05. CrdS-WT and CrdA∼P alone were used as controls (shown at right). (A) Phosphatase activity is shown for each mutant protein generated using site-directed mutagenesis. (B) Phosphatase activity of additional mutants generated within the highly conserved region. The N482K mutant (CA domain mutant) is shown as a control.

Our previous work demonstrated that CrdA can be phosphorylated *in vitro* by incubation with acetyl-phosphate (AcP) [4]. Radiolabeled CrdA∼P generated with [^32^P]AcP was used to assay phosphatase activity of CrdS WT and mutant proteins. CrdA∼P incubated with buffer alone was used to normalize the data (buffer control equal to 100% CrdA∼P; Figure 4). A five minute incubation was chosen because WT CrdS stimulated near complete loss of CrdA∼P (less than 2% remaining) at that time point. Of the CrdS alanine-scanning mutants tested, nine displayed a statistically significant reduction in CrdA∼P levels, as determined by a Student's *t*-test (*p<0.05; Figure 4). As expected, the H371A mutant displayed reduced phosphatase activity, which is similar to EnvZ from *E. coli* where the conserved histidine is not required but enhances phosphatase activity [32]. Seven other mutant proteins resulted in statistically significant reduction of CrdS phosphatase activity: V365A, R368A, L369A, I373A, P376A, P379A and I380A. Of these mutants, P376A and P379A displayed large decreases in CrdS phosphatase activity with greater than 50% of CrdA∼P remaining after a five minute incubation. This result was expected since proline substitutions should result in significant changes within the α helical structure and could possibly disrupt CrdS-CrdA interactions. However, these mutant proteins still retain kinase activity suggesting that the overall structure of these mutant proteins remains largely intact (Figure 2, Figure 3).

Of the mutants tested, only the CrdS-N375A mutant protein exhibited complete loss of phosphatase activity, with greater than 95% of CrdA∼P still present after five minutes. Additional substitution mutants were generated in CrdS-N375 to further assess the importance of this residue at this position. A conservative glutamine substitution, N375Q, also resulted in a mutant protein with no measurable phosphatase activity (Figure 4B). The N375D mutant substitutes an amide for an acidic group but also had no significant phosphatase activity. Lastly, the CrdS-N375T mutant protein was partially functional for phosphatase activity (50% CrdA∼P remaining), indicating some flexibility at this position, consistent with the observed sequence conservation at this position.

Of the nine mutant proteins affecting phosphatase activity, four are located within the highly conserved region surrounding the conserved histidine. The position E372, which is crucial for kinase activity, was not required for phosphatase activity as the E372A, E372D, E372G and E372Q substitutions all resulted in proteins fully competent for CrdA∼P dephosphorylation (Figure 4B). The I373A substitution resulted in a partial reduction in phosphatase activity as indicated by 28% of CrdA∼P still present after the five minute incubation. When the I373K, I373L and I373V mutant proteins were assayed for phosphatase activity they all possessed slightly increased levels of CrdA∼P compared to WT CrdS, although this increase was not statistically significant.

While the experiments detailed above assayed CrdA∼P levels after a five minute incubation with CrdS WT and mutant proteins, we wanted to further quantify the effect of CrdS mutants on CrdA∼P stability. To do this we determined the half-life of CrdA∼P when incubated with key CrdS mutant proteins. Phosphorylated CrdA is stable in the absence of CrdS with a half-life of 53.5 minutes (Table 1) [4]. Addition of WT CrdS reduces the CrdA∼P half-life to less than one minute, indicative of phosphatase activity (Table 1). The half-life of CrdA∼P was reduced to 2.6 minutes when incubated with CrdS-H371A, indicating that the conserved active site histidine minimally affects phosphatase activity. Additionally, the CrdS-E372A mutant protein, which has no kinase activity, retains WT phosphatase levels, as illustrated by a CrdA∼P half-life of 0.4 minutes. Conversely, the CrdS-N375A substitution mutant exhibited the most significant defect, with CrdA∼P displaying a half-life of 47.6 minutes, near that of CrdA∼P alone. This result indicates a complete loss of phosphatase activity for CrdS-N375A and highlights the critical role played by the conserved residue (N or T) at that position within HisKA proteins.

**Table 1:** Stability of CrdA∼P in the Presence of CrdS Mutant Proteins.

CrdS Protein	CrdA∼P Half Life (minutes)	SD	Reference
-	53.5	6.3	[4]
WT CrdS	0.7	0.3	[4]
H371A	2.6	0.5	This Study
E372A	0.4	0.1	This Study
N375A	47.6	3.2	This Study
E372A/N375A	50.2	0.7	This Study

The half-life of CrdA∼P was calculated when incubated with CrdS WT and mutant proteins. Values shown are the mean ± standard error. CrdA∼P was generated using [^32^P] acetyl-phosphate and incubated with equimolar amounts of CrdS mutant proteins. The half-life of CrdA∼P incubated alone is indicated in the top row. Mutations affecting CrdS phosphatase activity display a corresponding increase in CrdA∼P half-life.

### Phosphotransfer Assays with CrdS Phosphatase Mutants

**Figure 5:**
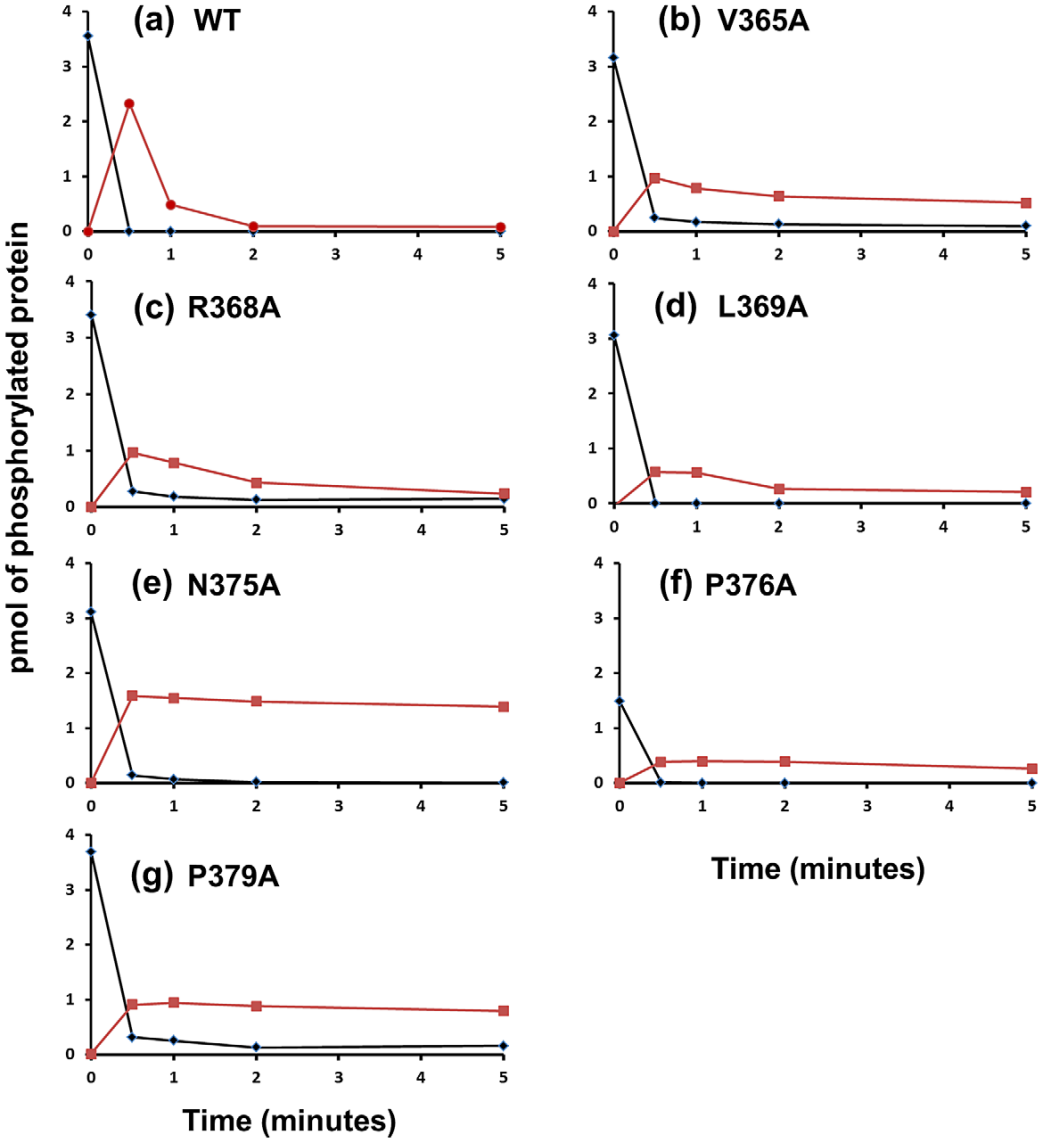
Phosphotransfer Assays with Phosphatase Mutants. CrdS-WT or mutant proteins displaying reduced CrdA∼P phosphatase activity were tested for phosphotransfer capacity. CrdS proteins were allowed to reach maximal phosphorylation and then mixed with equimolar amounts of CrdA. Samples were collected at 0, 0.5, 1, 2, and 5 minutes and resolved by electrophoresis, imaged and quantified. Results indicate pmol of phosphorylated proteins (y-axis) for (▪) CrdA∼P and (♦) CrdS∼P over time (x-axis). (A) WT CrdS (B) V365A (C) R368A (D) L369A (E) N375A (F) P376A (G) P379A.

Because phosphatase activity depends on protein interactions between the HK and RR∼P, we assayed the ability for the kinase competent, phosphatase deficient CrdS mutants (K+P−) to undergo phosphotransfer to CrdA. The capacity for such P− mutants to phosphorylate CrdA would allow us to conclude that the observed disruption in phosphatase activity was not merely due to defects in protein interactions between the HK and RR∼P. To test this, we assessed the ability of CrdS and various substitution mutants to undergo phosphotransfer to CrdA and subsequently dephosphorylate CrdA∼P *in vitro* (Figure 5). Therefore, this experiment allows for both phosphotransfer and phosphatase activities to be monitored concurrently.

We examined six CrdS kinase competent, phosphatase defective mutant proteins for their ability to undergo phosphotransfer and subsequent dephosphorylation of CrdA (Figure 5). Each mutant protein was able to autophosphorylate to near WT levels (>85% WT levels) with the exception of P376A. After each protein was allowed to reach maximal levels of phosphorylation (240 minutes autophosphorylation), CrdA was added in equimolar amounts. Upon addition of CrdA, WT CrdS∼P displayed fast phosphotransfer to CrdA and subsequent dephosphorylation of CrdA∼P (Figure 5). The majority of WT CrdS∼P was eliminated within one minute while CrdA∼P was still detectable at two minutes. In contrast, all six identified CrdS K+P− mutant proteins retained measurable CrdA∼P after a five minute incubation indicating that the mutant proteins V365A, R368A, L369A, N375A, P376A and P379A exhibit reduced phosphatase activity. The biggest defect in phosphatase activity was observed for the CrdS-N375A mutant protein where CrdA∼P levels did not decrease over the five minute time course (Figure 5e). Importantly, all six of these mutant proteins exhibited near WT phosphotransfer rates. These results indicate that K+P− mutant proteins are still able to interact with CrdA and that the resulting loss of phosphatase activity is not likely a result of decreased binding affinity for CrdA. Together these results allow us to conclude that CrdS-N375 is required exclusively for phosphatase activity.

### *In Vivo* Characterization of Kinase and Phosphatase Mutants of CrdS

In order to assess a role for CrdS kinase and phosphatase activities *in vivo* we first determined if various mutant proteins could complement the developmental phenotype of the *ΔcrdS* mutant. Previous work demonstrated that the *ΔcrdS* mutant has a severe aggregation and sporulation defect when spotted on CF starvation media and these defects could be complemented by ectopic expression of WT *crdS* [4]. Genes encoding the corresponding full-length CrdS (WT or mutant) proteins with an N-terminal T7-tag were introduced into the Mx8 phage attachment site and expressed using the constitutively active *pilA* promoter [33]–[35]. We generated constructs to express the WT (K+P+), E372A (K−P+), N375A (K+P−), and E372A/N375A (K−P−) mutant proteins within the *ΔcrdS* background. We utilized the E372A and N375A mutant proteins because they allow for separation of the corresponding kinase and phosphatase activities *in vitro*, as demonstrated above (Figure 3, Figure 4, Figure 5, Table 1). Additionally, we assayed the double E372A/N375A mutant protein which lacks both kinase and phosphatase activities (Figure 3, Figure 4). Immunoblot analysis indicated that each mutant CrdS protein is stably expressed under the conditions tested (Figure S5A).

**Figure 6:**
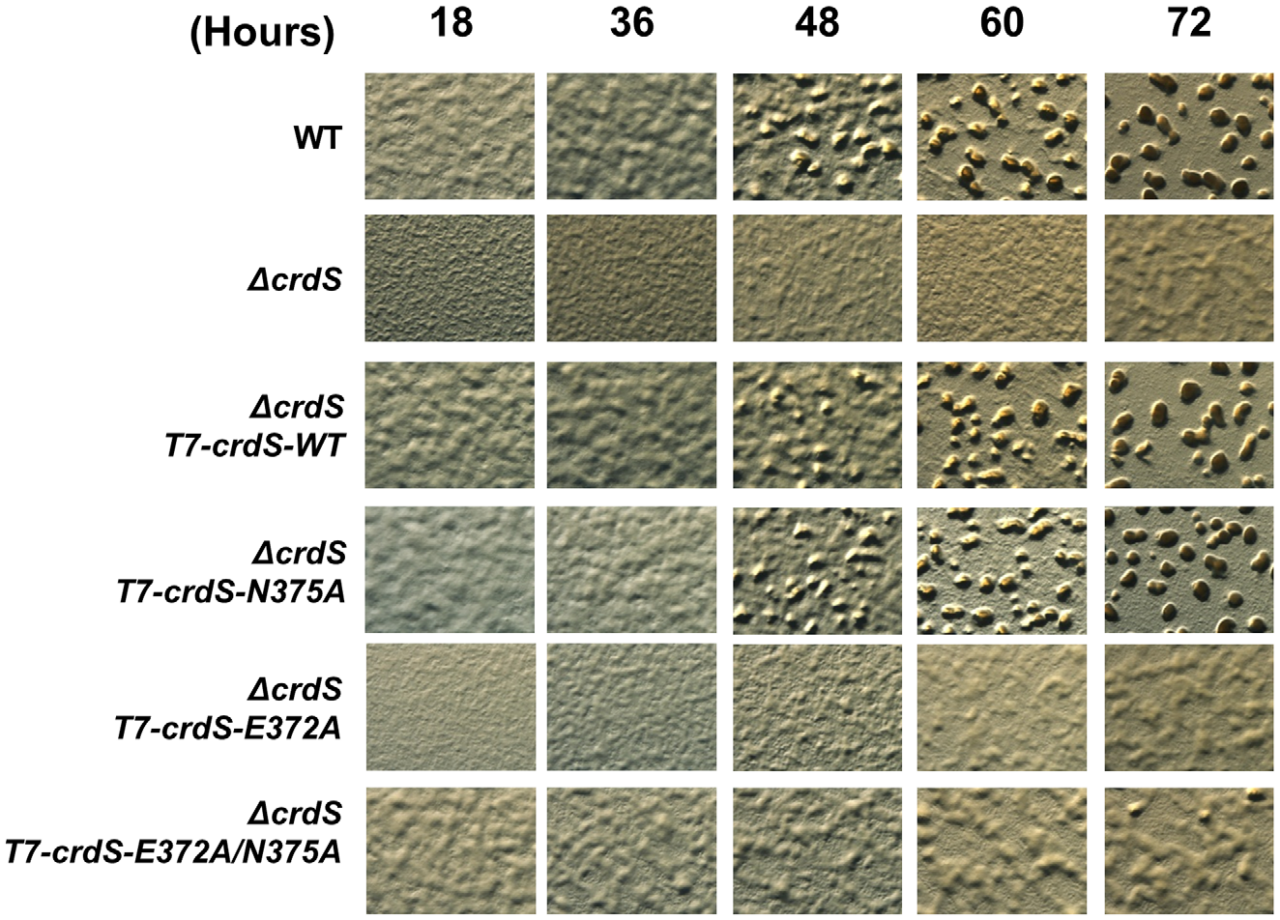
*M. xanthus* Development Is Affected by Expression of CrdS Kinase and Phosphatase Mutant Proteins. *M. xanthus* development was initiated by spotting cells on CF starvation media. CrdS-WT, E372A, N375A, and E372A/N375A mutant proteins were expressed in *ΔcrdS* cells using the constitutively active *pilA* promoter after stable integration at the Mx8 phage attachment site. Each picture is taken at 50× magnification at the time points indicated (in hours, top). *M. xanthus* development is evident by formation of phase dark aggregates which mature into fruiting bodies.

After 48 hours of incubation on starvation media, wild-type *M. xanthus* develops aggregation foci indicating the process of fruiting body formation is underway. Those foci darken by ∼72 hours indicating that cells have progressed through development to produce dormant myxospores (Figure 6). As we previously demonstrated, the *ΔcrdS* mutant cells display delayed developmental progression compared to WT with aggregation foci absent even at the 72 hour time point. Expression of *crdS-WT* in the *ΔcrdS* mutant restored WT timing with aggregation foci present by 48 hours, similar to WT cells. Likewise, expression of *crdS-N375A* restored aggregation to *ΔcrdS* mutant cells. In contrast, expression of *crdS-E372A* did not complement the *ΔcrdS* aggregation phenotype. This was expected since our previous results indicate that under starvation conditions CrdS shifts from acting as a phosphatase to acting as kinase (Figure 1) generating CrdA∼P to allow for expression of genes important for development. Thus, expression of the *crdS-E372A* mutant protein phenocopies the delayed aggregation phenotype of the *ΔcrdS* mutant (Figure 6). Unsurprisingly, expression of the *crdS-E372A/N375A* protein was unable to complement the *ΔcrdS* mutant indicating that the above results are attributable to the enzymatic activities for CrdS *in vivo*, rather than from incorporation of vector DNA or aberrant gene expression from the Mx8 phage attachment site. Together, these results indicate that CrdS kinase activity is required for developmental aggregation as depicted in our model in Figure 1.

The above complementation experiments do not fully discern the role for CrdS phosphatase activity *in vivo*. To assess this role, we performed a trans-dominance assay in the WT background using constructs that produce truncated, constitutively active forms of CrdS, as described previously [4]. Each *crdS* variant was expressed from the Mx8 phage attachment site using the *pilA* promoter with an N-terminal T7-tag replacing the input domain. When we expressed the K−P+ *crdS-E372A* allele, the role for CrdS phosphatase function became obvious: cells exhibited a severe delay in development where aggregation was not detectable until 72 hours and those aggregates remained translucent indicating that sporulation had not yet occurred (Figure S6). This result is strikingly similar to that obtained for the *ΔcrdS* mutant cells (Figure 6) and demonstrates that the phosphatase-competent mutant protein, CrdS-E372A, is dominant over the functional wild-type copy of *crdS*. In contrast, expression of *crdS-WT* and *crdS-N375A* resulted in premature development with aggregation foci appearing as early as 18 hours (Figure S6) [4]. These results indicate that CrdS phosphatase activity plays a role in proper developmental timing in *M. xanthus*.

Overall, our results illustrate that kinase and phosphatase functions are genetically separable for CrdS and depend on specific residues within the DHp domain, namely E372 for kinase activity and N375 for phosphatase activity. These results are consistent with the model depicted in Figure 1, whereby a shift from phosphatase to kinase activity is critical for CrdSA regulation of aggregation during *M. xanthus* development.

### Residues Required for Kinase and Phosphatase Function Are Conserved in Other HisKA Proteins

**Figure 7:**
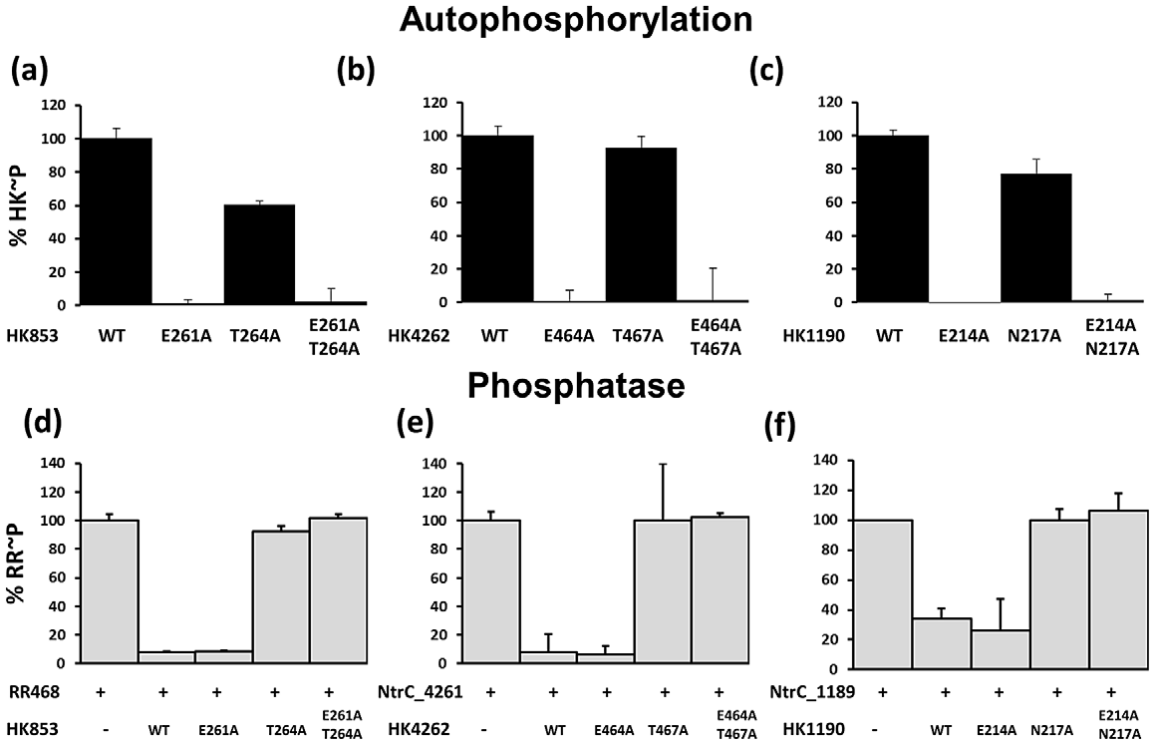
Kinase and Phosphatase Assays with HisKA Proteins *T. maritima* HK853 and *M. xanthus* HK4262 and HK1190. (a–c) *In vitro* kinase assays with purified protein. Each kinase was allowed to phosphorylate for 30 minutes and the amount of WT phosphorylated protein was set to 100%. (a) HK853 from *T. maritima* (b) HK4262 from *M. xanthus* (c) HK1190 from *M. xanthus*. (d–f) Phosphatase assays using acetyl-phosphate labeled response regulators. The amount of phosphorylated RR incubated in buffer alone was set to 100%. (d) *T. maritima* RR468 and HK853 (e) *M. xanthus* NtrC4261 and HK4262 (f) *M. xanthus* NtrC1189 and HK1190. Substitution mutants utilized here correspond to residues within the HisKA conserved sequence H-E/D-xx-T/N.

Our *in vitro* and *in vivo* assays demonstrate that the conserved E372 residue within CrdS is required for kinase function and N375 is required for phosphatase function. Because these residues are highly conserved within the HisKA subfamily, we made similar mutations in three other related HKs; *M. xanthus* HK1190, *M. xanthus* HK4262 and *T. maritima* HK853 (Figure 2D, Figure 7A). *M. xanthus* HK1190 and HK4262 are two HisKA subfamily kinases which are competent for *in vitro* autophosphorylation and subsequent phosphotransfer to their cognate response regulators [4]. We also assayed HK853 from *T. maritima* which arguably has the best structural data for any bacterial HK. Both *T. maritima* HK853 and *M. xanthus* HK4262 have a Thr rather than Asn at the phosphatase position, as compared to CrdS, consistent with the observed flexibility in the consensus sequence. HK853, HK4262 and HK1190 each contain Glu at the acidic residue position corresponding to E372 of CrdS. Purification of His-tagged versions of all wild type and mutant forms yielded soluble proteins.

Wild type versions of HK853, HK4262 and HK1190 each displayed kinase activity as evidenced by incorporation of [γ-^32^P]ATP (Figure 7A–7C). The amount of phosphorylation for each WT protein observed at the 30 minute time point was set to 100%. To test the hypothesis that the residue adjacent to the conserved His is required for kinase activity in these HisKA homologs, we assayed the HK853-E261A, HK4262-E464A and HK1190-E214A mutants for kinase activity. Each protein displayed an inability to autophosphorylate, similar to the CrdS-E372A mutant protein. Additionally, mutants predicted to affect phosphatase function exclusively, HK853-T264A, HK4262-T467A and HK1190-N217A, were each found to be competent for autophosphorylation with levels near that of their respective WT kinases (Figure 7).

Phosphatase activity was directly assayed by measuring dephosphorylation of acetyl-phosphate labeled RRs for each TCS cognate pair: HK853-RR468, HK4262-NtrC4261, and HK1190-NtrC1189. Each response regulator has been shown previously to be stably phosphorylated using AcP [4], [23]. RR∼P levels following incubation in buffer were arbitrarily set at 100% (Figure 7D–7F).) [4], [23]. As predicted, incubation of each WT HK with its cognate RR∼P resulted in loss of phosphorylation for the regulator. Similar to the CrdS-E372A mutant, incubation of the HK853-E261A, HK4262-E464A and HK1190-E214A kinase defective mutants with their cognate RR also resulted in WT levels of phosphatase activity, indicating these residues are not required for phosphatase activity (Figure 7).

Based on our results above for CrdS, we predicted that the conserved residues HK853-T264, HK4262-T467 and HK1190-N217 would be required for phosphatase activity. The results show that the HK853-T264A, HK4262-T467A and HK1190-N217A proteins exhibited a near complete loss of phosphatase activity (∼100% RR∼P levels remaining) relative to the controls (Figure 7D–7F), while retaining kinase activity (Figure 7A–7C). Together, the results indicate that the conserved acidic residue (E/D) and the conserved hydroxyl/amide residue (T/N) serve similar functions in kinase and phosphatase activity, respectively, within the HisKA subfamily of TCS kinases.

## Discussion

### Summary

In this study we performed alanine-scanning mutagenesis of the α1 helix in the HisKA domain of CrdS in order to determine a role for each residue in kinase and phosphatase function. We chose α1 within the HisKA domain because it contains the phosphorylatable histidine residue and serves as the major interaction domain with its cognate RR, CrdA, based on structural and covariance studies from other TCS [23], [24]. Sequence analysis by us and other groups revealed a highly conserved region adjacent to the phosphorylated His residue (Figure 2), suggesting that these residues are likely involved in either kinase or phosphatase function for HisKA subfamily kinases [12], [28].

**Figure 8:**
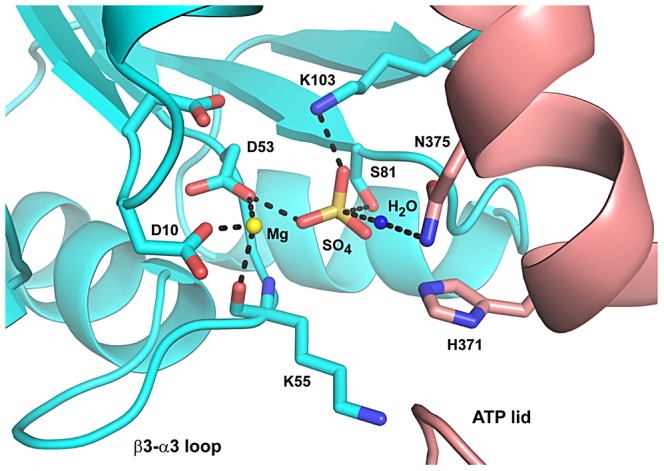
Proposed Mechanism for CrdA∼P Dephosphorylation by CrdS. This model depicts the proposed dephosphorylation reaction of CrdA∼P by CrdS and is adapted from the HK853-RR468 structure determined by Casino *et al.* [23]. Residues are depicted in cyan for CrdA and in salmon for CrdS. A magnesium ion is shown as a yellow sphere and sulfate represents a phosphoryl group of CrdA. Dotted lines represent predicted ionic or hydrogen bonds between atoms in close proximity.

All CrdS mutant proteins were subjected to a series of *in vitro* assays that measured autokinase function, phosphotransfer rates to the target regulator CrdA, and phosphatase activity toward CrdA∼P. Several CrdS mutants were identified for defects in kinase or phosphatase function and key mutants were further tested for defects *in vivo*. Together the results lead to the conclusion that only a single residue, N375, is required exclusively for CrdS phosphatase activity. In order for N375 to play a critical role in phosphatase activity, the residue would need to coordinate a water molecule to allow for nucleophilic attack of the phosphoryl group on CrdA-D53, as has been proposed for HK853-RR468 in *T. maritima*. Thus, we built a structural model for the CrdS-CrdA interaction (Figure 8) based on the *T. maritima* HK853-R468 co-structure [23]. The resulting co-structure demonstrates that CrdS-N375 is in proper register to allow for coordination of a water molecule for nucleophilic attack on the phospho-aspartate residue of CrdA.

Additionally, we were able to identify a mutation, CrdS-E372A, adjacent to the conserved histidine, which abolished kinase activity without affecting phosphatase activity. We also determined positions in the highly conserved region of the DHp α1 helix of CrdS that appeared to tolerate substitutions. *In vivo* expression of key mutant proteins (E372A and N375A) provided evidence that CrdS kinase and phosphatase activity are both important for CrdSA regulation of timing of development in *M. xanthus*. Finally, mutations in the corresponding residues in HK853 from *T. maritima* as well as HK4262 and HK1190 of *M. xanthus* each displayed defects in kinase and phosphatase activity *in vitro*, similar to those seen in CrdS. Together, the results indicate the importance of these conserved residues for kinase and phosphatase function of HisKA proteins.

### A Conserved Residue Required for Kinase Activity

A general strategy for studying bacterial HKs is to generate His to Ala mutants in the conserved site of autophosphorylation. While this can be useful for discerning a role for phosphorylation within a given two-component signaling system, it is important to realize limitations with this method. For instance, the CrdS-H371A mutant abolishes kinase activity while displaying a modest reduction in phosphatase activity (Figure 4, Table 1). In contrast, mutation of the conserved histidine in *Caulobacter crescentus* PleC (H610A) resulted in a loss of both kinase and phosphatase activities, highlighting the variable requirement of this residue for phosphatase function in some proteins [36].

In the present study, substitutions in only one amino acid, CrdS-E372, resulted in a mutant with no measurable kinase activity while retaining full phosphatase activity (Figure 3, Figure 4). The E372 residue is adjacent to the conserved site of phosphorylation, H371, and likely plays a role in generating a suitable environment for His phosphorylation. Sequence alignments show this position is highly conserved within the HisKA subfamily of sequences (Figure 2). CrdS-E372A, E372D, E372G and E372Q mutant proteins each lacked kinase activity indicating a strict requirement of this amino acid for proper CrdS kinase function (Figure 3). Furthermore the *T. maritima* HK853-E261A, *M. xanthus* HK4262-E464A and *M. xanthus* HK1190-E214A mutant proteins were also kinase deficient, yet phosphatase competent proteins. This result is similar to those for *E. coli* NtrB where the corresponding NtrB-E140A and NtrB-E140Q proteins also resulted in phenotypes consistent with a K− protein [37]. The *crdS-E372A* allele was unable to complement the *ΔcrdS* mutant (Figure 6, Figure S5) demonstrating the importance of kinase activity for CrdS function *in vivo* [4]. Overall our results illustrate the importance of the acidic residue adjacent to the conserved histidine for the proper kinase function for the HisKA subfamily of proteins.

### A Conserved Residue Required for HisKA Phosphatase Activity

Recent work by Huynh *et al.* on a HisKA_3 protein, NarX, demonstrated that two residues in the highly conserved region of the DHp α1 helix (DxxxQ), adjacent to the conserved His residue, are required for phosphatase activity [12]. Similarly, two residues within the *Borrelia burgdorferi* CheX phosphatase (ExxN) are required to dephosphorylate its target response regulator, CheY [7], [38]. Furthermore, CheC and CheZ phosphatases, also contain a two amino acid motif required for phosphatase activity [38]. Thus, Huynh *et al.* proposed that the corresponding two residues in the HisKA subfamily would be required for phosphatase activity.

Based on our results here, we conclude that only one residue corresponding to CrdS-N375 is required exclusively for phosphatase activity in the HisKA family proteins. Our results indicate that CrdS-E372 is not required for phosphatase activity, since the CrdS-E372A, E372D, E372G and E372Q mutants all resulted in phosphatase competent proteins (Figure 4). Additionally, the corresponding mutant proteins *T. maritima* HK853-E261A, *M. xanthus* HK4262-E464A and *M. xanthus* HK1190-E214A displayed wild-type phosphatase activity *in vitro* (Figure 7). Similarly, previous studies on NtrB indicated the corresponding acidic residue is not likely required for phosphatase activity as the NtrB-E140A and NtrB-E140Q mutants retained a phosphatase competent phenotype, although no *in vitro* biochemistry has been performed to confirm this [37]. Therefore, we conclude the acidic residue adjacent to the conserved histidine is important for kinase activity in HisKA proteins, but is not required for phosphatase activity.

The CrdS-N375A substitution mutant was unable to dephosphorylate CrdA∼P in an assay directly measuring phosphatase activity (Figure 4). Similar mutations in three HKs, *T. maritima* HK853, *M. xanthus* HK1190 and HK4262 each resulted in a loss of phosphatase activity (Figure 7). These results are comparable to those reported for other HisKA subfamily kinases, EnvZ, ResE, PleC, CpxA, PhoR and WalK where single mutations at the residue corresponding to CrdS-N375 abolished phosphatase activity [10], [11], [19], [25], [39], [40]. In addition, the co-structure of HK853-RR468 provided support for the role of the conserved Thr/Asn in HisKA proteins where this residue does not contact the RR, but is positioned to coordinate a water molecule for nucleophilic attack on the phospho-aspartate [23]. Taken together, these studies indicate the critical role for the conserved hydroxyl/amide moiety (Thr/Asn) for phosphatase activity within HisKA proteins.

The observation that only a single residue is sufficient for phosphatase activity is not unique and was recently described for the RapH phosphatase in *Bacillus subtilis*. The RapH phosphatase directly dephosphorylates Spo0F, a stand-alone receiver protein, central to the phosphorelay system controlling sporulation in *B. subtilis*. The amide moiety on residue RapH-Q47 is sufficient to coordinate a water molecule for nucleophilic attack on phosphorylated Spo0F [9]. Thus we hypothesize a similar requirement for HisKA family proteins where the conserved Thr/Asn is sufficient to coordinate the nucleophilic water molecule for dephosphorylation of the RR (Figure 8).

### Concluding Remarks

CrdS interacts with the Che3 chemosensory system as part of a complex signal transduction pathway to regulate phosphorylation of the response regulator, CrdA [4]. Complex regulatory cascades provide multiple inputs and checkpoints for regulation of complex processes such as sporulation in *B. subtilis* and *M. xanthus* [41]. The results in this study identified residues in CrdS that modulate kinase and phosphatase activity to control developmental aggregation in *M. xanthus*.

Our work defines the function of conserved residues within the α1 helix of the HisKA domain of CrdS. The substitution in E372 resulted in a CrdS mutant which could not function as a kinase, but could function as a phosphatase. Conversely, substitutions in N375 resulted in a form of CrdS that was capable of kinase activity, but lacked phosphatase function. Similar results were obtained for two additional *M. xanthus* HKs, HK1190 and HK4262, as well as for *T. maritima* HK853. Our results are consistent with the data published on numerous HisKA domain containing kinases including EnvZ, ResE, PleC, CpxA, PhoR and WalK [10], [11], [19], [25], [39], [40]. These results indicate that the conserved Thr/Asn residue is required exclusively for phosphatase activity while the conserved acidic residue adjacent to the site of phosphorylation is required exclusively for kinase activity in HisKA proteins. Collectively, these studies suggest that HisKA proteins, the dominant member of HKs in bacteria, utilize common mechanisms for kinase or phosphatase function.

## Materials and Methods

### Bacterial Growth and Strain Construction

All strains used in this study are detailed in Table S1. *E. coli* strains were grown in LB with antibiotic concentrations of 40 µg/mL kanamycin, 15 µg/mL oxytetracycline or 100 µg/mL ampicillin when selection was required. For routine growth of *M. xanthus*, cells were grown in CYE (10 mM MOPS pH 7.6, 1% w/v Casitone, 0.5% Yeast Extract, 4.0 mM MgSO_4_) with 80 µg/mL kanamycin and 7.5 µg/mL oxytetracycline added when required. All strains were constructed using standard cloning techniques (see Table S1 for strains). Site-directed mutagenesis was performed using the QuikChange kit from Agilent (see Table S2 for primers). All point mutations were verified by sequencing. Proteins were expressed using *E. coli* BL21(DE3) from the IPTG (isopropyl-β-d-thiogalactopyranoside)-inducible vector, *pET28a* (Novagen). For *M. xanthus* developmental assays, cells were grown in CYE to a density of 150 klett units (KU). 10 µL was then spotted on CF developmental media and allowed to grow at 32°C [3]. Pictures were taken at indicated times with a Nikon SMZ1500 microscope using QImaging MicroPublisher 5.0 RTV CCD camera, processed with QCapture Pro software and edited in Microsoft PowerPoint 2011.

### Sequence Alignments, Comparisons, and Protein Modeling

All sequence logos were generated using Weblogo [30]. The sequence Weblogo of HisKA domains in 2D was generated using BLAST-P to identify the 100 most diverse HisKA domains as identified using the NCBI database (See Table S3 for complete list) [29]. While *M. xanthus* has 144 annotated HKs only 122 were included in this analysis, because the remaining 24 putative HKs have no annotated DHp domains as determined by the MiST signal transduction database [2]. Of the resulting 122 HK's, 118 have DHp domains of the HisKA subfamily. The sequence logo in Figure S1 was generated using 118 canonical *M. xanthus* HisKA containing kinases as obtained from the MiST Database. Sequence alignments in Figure 2C were generated using ClustalW (DNASTAR v8 Lasergene). For a list of sequence accession numbers as well as amino acids shown in Figure 2C, see Table S4.

The structure of CrdS was generated using Phyre to model CrdS on known structure of HK853 from *T. maritima* [42]. Figure 8 is adapted from Figure 2E of Casino et al. [23]. The CrdA response regulator (residues 1–120) and histidine kinase CrdS (residues 347–578) were modeled by the Phyre^2^ server using PDB entry 3DGE as template [23], [43]. Images were generated using PyMol (DeLano Scientific).

### Protein Purification

A truncated form of CrdS (expressing amino acids 346–578) was used for all *in vitro* assays, where the insoluble N-terminal membrane spanning domain is replaced by a 6x-His affinity tag [4]. All proteins were expressed using the IPTG (isopropyl-β-d-thiogalactopyranoside) inducible vector pET28a. For overexpression, 1 L of Luria Broth was inoculated with 25 mL of overnight culture and grown at 37°C with shaking until the OD_600_ (optical density at 600 nm) reached 0.4 to 0.6. Cultures were then induced by addition of 0.5 mM IPTG and grown overnight at 20°C, pelleted by centrifugation then stored at −20°C until purification.

All *M. xanthus* proteins were purified using a batch purification method. Frozen cell pellets were resuspended in 10 mL Lysis buffer (25 mM Tris pH 7.6, 125 mM NaCl, mini-EDTA Free protease inhibitor from Roche) and lysed using CelLytic (Sigma). Lysates were clarified by spinning at 50,000×*g* and added to 2 mL His-select Cobalt affinity gel (Sigma) equilibrated with Lysis buffer. Samples were incubated at 4°C for 2 hours with mild shaking. The resin was pelleted with a one minute spin at 100×*g* and washed 3 times with 10 mL Lysis buffer. Protein was eluted using Elution buffer (25 mM Tris pH 7.6, 125 mM NaCl, 500 mM imidazole) and dialyzed overnight against one liter of Dialysis buffer (25 mM Tris pH 7.6, 125 mM NaCl, 1 mM DTT, 1% Triton X100, 50% glycerol). Samples were stored at −20°C until use. Sample purity was assessed using Coomassie stained SDS-PAGE gels. Protein concentrations were determined using Bradford Reagent (BioRad). Detailed purification protocols for HK853 and RR468 are provided in Text S1.

### *In Vitro* Biochemical Assays

Autokinase, phosphotransfer, phosphatase and half-life calculations were performed following previously described experimental protocols [4]. For kinase reactions, each HK (5 µM) was incubated in 1× kinase buffer (25 mM Tris pH 7.6, 50 mM KCl, 1 mM of CaCl_2_ MgCl_2_ and MnCl_2_, 1 mM β-mercaptoethanol) and ATP mix (250 µM ATP, 0.3 µM [γ-^32^P]ATP). Reactions were allowed to proceed for 1, 5, 30, 60, and 240 minutes before reactions were stopped by addition to an equal volume of SDS-loading buffer. After samples were separated by SDS-PAGE, gels were exposed to phosphor screens and quantified using ImageQuant v5.1. Reactions were compared to a standard curve generated using [γ-^32^P]ATP of known quantities, with bar graphs showing a representative data set.

Phosphatase experiments were carried out using RR∼P phosphorylated using radiolabeled acetyl phosphate ([^32^P]AcP; Text S1) [4], [44]. *M. xanthus* RR∼P were incubated for 5 minutes with corresponding kinase proteins. Phosphatase assays with *T. maritima* RR468∼P utilized a 30 minute time point for dephosphorylation by HK853. RR∼P incubated with buffer alone was set to 100%. Samples were separated by SDS-PAGE and gels were exposed to phosphor screens and visualized using a Typhoon Imager. ImageQuant v5.1 was used to determine integrated pixel density and subtract background. Bar graphs shown are average of three experiments with standard deviation.

### *In Vivo* Expression of CrdS Mutant Proteins

*M. xanthus* CrdS expression constructs were generated as previously described [4]. Briefly, *crdS* point mutants were subcloned from pET28 vectors into the Mx8 integration plasmid pWB200 under control of the constitutive *pilA* promoter. The expressed version of CrdS contained the T7-tag which allowed for western blot analysis. Plasmids were then incorporated into the Mx8 phage attachment site and verified by PCR.

### Western Blot Analysis

To confirm stable expression of CrdS mutant protein under conditions tested, western blot analysis was performed. 100 µL of a 100 KU suspension of cells was spread plated on CF minimal media plates. After growth for 3 days, cells scraped from the plate and washed in (WLB) Western lysis buffer (10 mM Tris pH 7.6, 125 mM NaCl, 1 mM EDTA, 1 mini-Protease inhibitor tablets from Roche per 10 mL of solution). Cell pellets were then stored at −20 C until Western blot analysis was performed. Cells pellets were resuspended in 250 µL WCB, lysed by sonication and protein concentration was determined using Bradford Reagent. 1 µg of cell solution was run on a 12% SDS-PAGE gel, and transferred to PVDF membrane, blocked overnight, and proteins were visualized using α-T7 antibody (Pierce).

### Circular Dichroism Spectroscopy

Protein samples for CD (Circular Dichroism) spectroscopy were purified as described above and then dialyzed overnight in CD buffer (25 mM Na Phosphate 7.6, 50 mM NaCl). Protein concentration was determined and samples diluted to a final concentration of 25 µM in CD buffer. Samples were then analyzed using a 1 mM cuvette in a Jasco J-815 CD spectrometer. Data shown is average of three spectral scans.

## Supporting Information

Figure S1 Sequence conservation within the α1 helices of all 118 *M. xanthus* HisKA DHp domains and numbered according to residue position within CrdS.
(TIF)

Figure S2 Coomassie of Purified Proteins: SDS-PAGE gels of CrdA and CrdS mutant proteins purified and used within this paper. Samples were run on four separate gels and merged using PowerPoint. CrdA has an apparent molecular weight of 57 kDa, while CrdS has an approximate molecular weight of 27 kDa.
(TIF)

Figure S3 Circular Dichroism Spectra of CrdS Mutant Proteins. CD spectra were determined as described in *Materials and Methods*. Spectra shown are the average of three scans. The data indicates CrdS substitution mutant proteins have the same secondary structure as wild type CrdS.
(TIF)

Figure S4 Autophosphorylation Curves of Key Point Mutants: The amount of phosphorylated (pmol) CrdS-WT, CrdS-H371A, CrdS-E372A, and CrdS-N375A was measured as described in *Materials and Method*s. CrdS-WT and CrdS-N375A are the only proteins displaying kinase activity after 4 hours.
(TIF)

Figure S5 Western Blot Analysis to Verify Stable Expression of CrdS proteins in *M. xanthus*. CrdS WT and mutant proteins were expressed after stable integration at the Mx8 phage attachment site using the constitutively active *pilA* promoter. Each protein encodes an N-terminal T7-tag. Equal amounts of protein are loaded in each lane and proteins are visualized using a α-T7 antibody. (a) Western blot analysis of proteins expressed in Figure 6. (b) Western blot analysis of proteins expressed in Figure S6.
(TIF)

Figure S6 Development of *M. xanthus* is Affected by Expression of CrdS Kinase and Phosphatase Mutant Proteins Expressed in the WT Background. Trans-dominance experiments were conducted by expressing a truncated constitutively active form of *crdS* in WT *M. xanthus* cells. CrdS WT, E372A, N375A, and E372A/N375A mutant proteins were expressed using the constitutively active *pilA* promoter after stable integration at the Mx8 phage attachment site. Each picture is taken at 50× magnification at the time points indicated (in hours, top). *M. xanthus* development is indicated by formation of phase dark aggregates termed fruiting bodies.
(TIF)

Table S1 Stain list detailing strains used and generated in this study.
(DOCX)

Table S2 Primers used in this study.
(DOCX)

Table S3 List of 100 Most Diverse HisKA domains. GI numbers of 100 most diverse HisKA domains as determined by BLAST. Data were used to make the sequence logo depicted in Figure 2D.
(DOCX)

Table S4 List of protein sequences and Accession numbers. List of protein sequences and accession numbers of proteins used to generate Figure 2C
(DOCX)

Text S1 Supplemental Materials and Methods: Purification protocols for *Thermotoga maritima* proteins and for synthesis of radiolabeled acetyl-phosphate.
(DOCX)
