# Supplementary figures and images for: Genetic and Biochemical Dissection of a HisKA Domain Identifies Residues Required Exclusively for Kinase and Phosphatase Activities
Source: PLoS Genet. 2012 Nov 29;8(11):e1003084. doi: 10.1371/journal.pgen.1003084 (PMC3510030; doi:10.1371/journal.pgen.1003084)

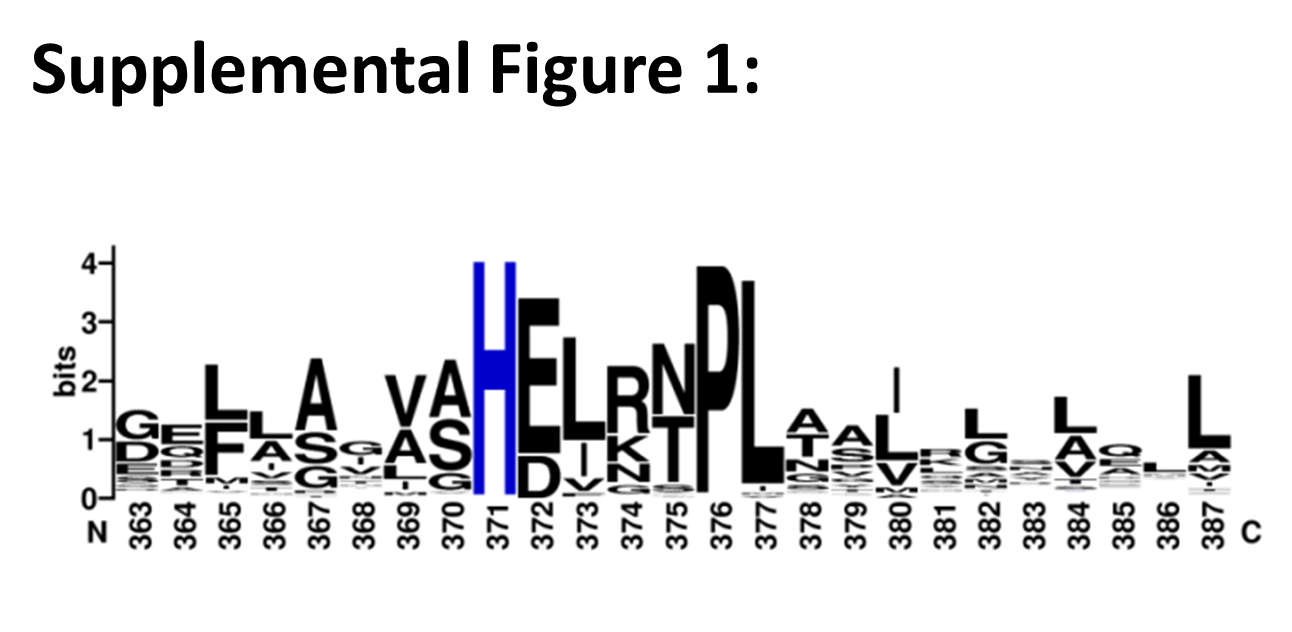

Supplement: Figure S1 — Sequence conservation within the α1 helices of all 118 M. xanthus HisKA DHp domains and numbered according to residue position within CrdS. (TIF) [file pgen.1003084.s001.tif]

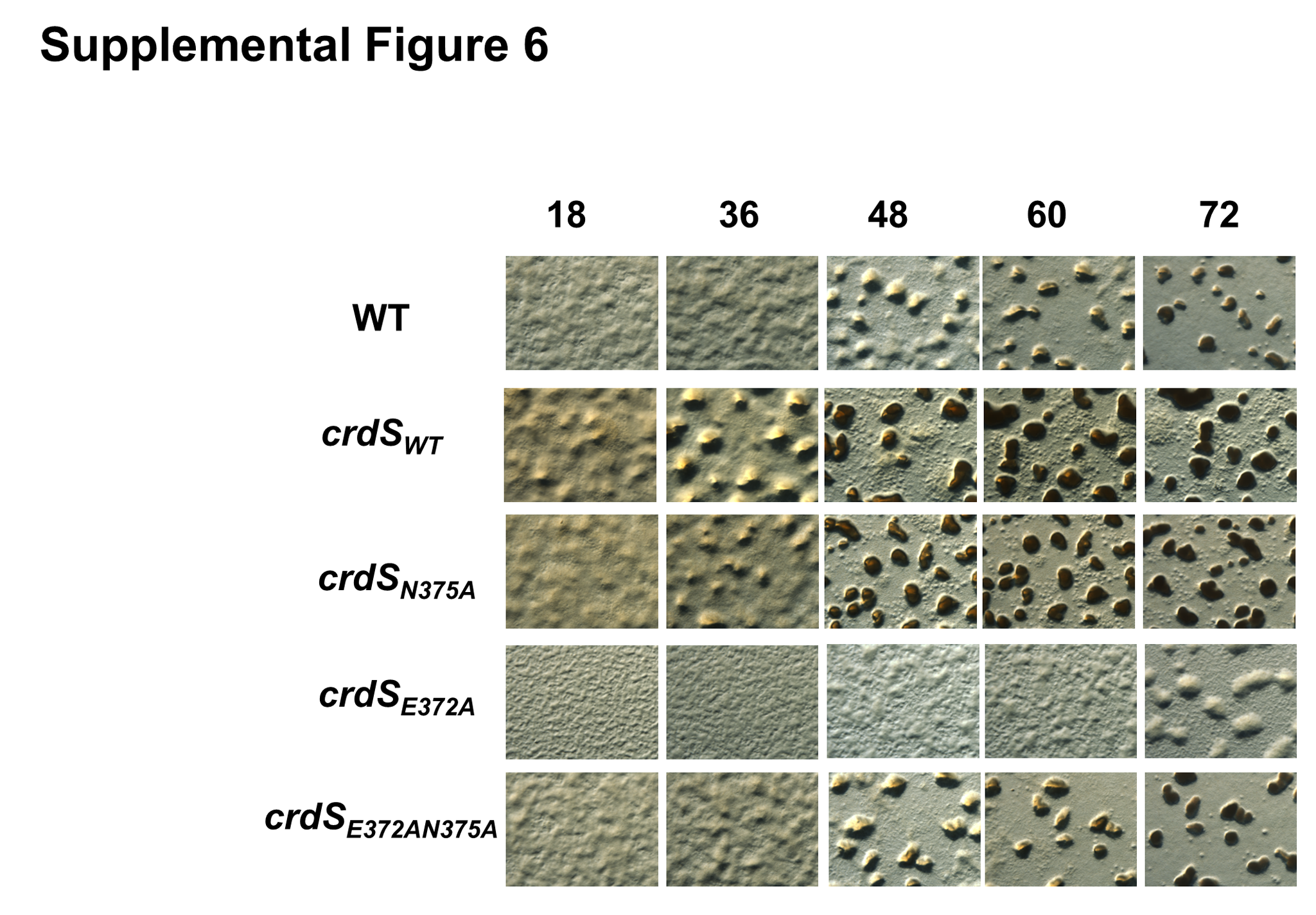

Supplement: Figure S6 — Development of M. xanthus is Affected by Expression of CrdS Kinase and Phosphatase Mutant Proteins Expressed in the WT Background. Trans-dominance experiments were conducted by expressing a truncated constitutively active form of crdS in WT M. xanthus cells. CrdS WT, E372A, N375A, and E372A/N375A mutant proteins were expressed using the constitutively active pilA promoter after stable integration at the Mx8 phage attachment site. Each picture is taken at 50× magnification at the time points indicated (in hours, top). M. xanthus development is indicated by formation of phase dark aggregates termed fruiting bodies. (TIF) [file pgen.1003084.s006.tif]
