# Supplementary material for: Genetic and Biochemical Dissection of a HisKA Domain Identifies Residues Required Exclusively for Kinase and Phosphatase Activities
Source: PLoS Genet. 2012 Nov 29;8(11):e1003084. doi: 10.1371/journal.pgen.1003084 (PMC3510030; doi:10.1371/journal.pgen.1003084)
Supplement: Table S1 — Stain list detailing strains used and generated in this study. (DOCX) [file pgen.1003084.s007.docx]

**Supplemental Table 1:** **Stain list detailing strains used and generated in this study**

| **Strain #** | **Strain** | **Reference** |
| --- | --- | --- |
| DZ2 | Wild-type *Myxococcus xanthus* | [4] |
| JK1857 | *DZ2 pWB200::PpilA::T7-crdS* | [4] |
| JK1867 | *BL21(DE3) pET28::ntrC_1189* | [4] |
| JK1869 | *BL21(DE3) pET28::ntrC_4261* | [4] |
| JK1868 | *BL21(DE3) pET28::HK1190* | [4] |
| JK1870 | *BL21(DE3) pET28::HK4262* | [4] |
| JK1864 | *BL21(DE3) pET28::crdA* | [4] |
| JK1862 | *BL21(DE3) pET28::crdS(Wild-Type)* | [4] |
| JK1863 | *BL21(DE3) pET28::crdS(H371A)* | [4] |
| JK3514 | *BL21(DE3) pET28::crdS(E372A)* | This Study |
| JK3516 | *BL21(DE3) pET28::crdS(I373A)* | This Study |
| JK3518 | *BL21(DE3) pET28::crdS(K374A)* | This Study |
| JK3520 | *BL21(DE3) pET28::crdS(P376A)* | This Study |
| JK3522 | *BL21(DE3) pET28::crdS(A370S)* | This Study |
| JK3524 | *BL21(DE3) pET28::crdS(L369A)* | This Study |
| JK3526 | *BL21(DE3) pET28::crdS(R368A)* | This Study |
| JK3528 | *BL21(DE3) pET28::crdS(V365A)* | This Study |
| JK3530 | *BL21(DE3) pET28::crdS(T378A)* | This Study |
| JK3532 | *BL21(DE3) pET28::crdS(T386A)* | This Study |
| JK3544 | *BL21(DE3) pET28::crdS(E372A, N375A)* | This Study |
| JK3546 | *BL21(DE3) pET28::crdS(E372D)* | This Study |
| JK3548 | *BL21(DE3) pET28::crdS(K374E)* | This Study |
| JK3550 | *BL21(DE3) pET28::crdS(N375A)* | This Study |
| JK3552 | *BL21(DE3) pET28::crdS(L377A)* | This Study |
| JK3554 | *BL21(DE3) pET28::crdS(Q363A)* | This Study |
| JK3556 | *BL21(DE3) pET28::crdS(R367A)* | This Study |
| JK3558 | *BL21(DE3) pET28::crdS(I380A)* | This Study |
| JK3560 | *BL21(DE3) pET28::crdS(M382A)* | This Study |
| JK3562 | *BL21(DE3) pET28::crdS(L387A)* | This Study |
| JK3564 | *BL21(DE3) pET28::crdS(N482K)* | This Study |
| JK3566 | *BL21(DE3) pET28::crdS(I373K)* | This Study |
| JK3568 | *BL21(DE3) pET28::crdS(N375T)* | This Study |
| JK3581 | *BL21(DE3) pET28::CrdS(N375Q)* | This Study |
| JK3602 | *BL21(DE3) pET28::crdS(E364A)* | This Study |
| JK3604 | *BL21(DE3) pET28::crdS(A366S)* | This Study |
| JK3606 | *BL21(DE3) pET28::crdS(S383A)* | This Study |
| JK3609 | *BL21 pET28::crdS(R381A)* | This Study |
| JK3636 | *BL21(DE3) pET28::crdS(K374R)* | This Study |
| JK3637 | *BL21(DE3) pET28::crdS(P379A)* | This Study |
| JK3638 | *BL21(DE3) pET28::crdS(L384A)* | This Study |
| JK3639 | *BL21(DE3) pET28::crdS(E385A)* | This Study |
| JK3640 | *BL21(DE3) pET28::crdS(E372Q)* | This Study |
| JK3641 | *BL21(DE3) pET28::crdS(E372G)* | This Study |
| JK3642 | *BL21(DE3) pET28::crdS(I373L)* | This Study |
| JK3643 | *BL21(DE3) pET28::crdS(I373V)* | This Study |
| JK3645 | *BL21(DE3) pET28::crdS(H371A,N482K)* | This Study |
| JK3663 | *DH5α pWB200::PpilA::T7::crdS(H371A)* | This Study |
| JK3664 | *DH5α pWB200::PpilA::T7::crdS(E372A,N375A)* | This Study |
| JK3666 | *DH5α pWB200::PpilA::T7::crdS(N375A)* | This Study |
| JK3670 | *DH5α pWB200::PpilA::T7::crdS(E372A)* | This Study |
| JK3680 | *BL21(DE3) pET28::HK_4262 (E484A)* | This Study |
| JK3681 | *BL21(DE3) pET28::HK_4262 (T487A)* | This Study |
| JK3701 | *BL21(DE3) pET28::HK_4262 (E484A/T487A)* | This Study |
| JK3703 | *DZ2 Mx8::pWB200::PpilA::T7::crdS(N375A)* | This Study |
| JK3705 | *DZ2 Mx8::pWB200::PpilA::T7::crdS(E372A)* | This Study |
| JK3711 | *BL21(DE3) pET28::HK_1190(E214A)* | This Study |
| JK3712 | *BL21(DE3) pET28::HK_1190(N217A)* | This Study |
| JK3713 | *BL21(DE3) pET28::HK_1190(E214A/N217A)* | This Study |
| JK3716 | *BL21(DE3) pET28::HK853* | This Study |
| JK3717 | *Bl21(DE3) pET28::RR468* | This Study |
| JK3722 | *DZ2 Mx8::pWB200::PpilA::T7::crdS(E372A/N375A)* | This Study |
| JK3760 | *BL21(DE3) pET28::HK853 (E261A)* | This Study |
| JK3761 | *BL21(DE3) pET28::HK853 (T264A)* | This Study |
| JK3762 | *BL21(DE3) pET28::HK853 (E261A/T264A)* | This Study |
| JK3764 | *BL21 (DE3) pET28::NtrC_5153 (Rec)* | This Study |
| JK3766 | *BL21 (DE3) pET28::NtrC_1189 (Rec)* | This Study |
| JK3768 | *BL21 (DE3) pET28::NtrC_4261 (Rec)* | This Study |
