# Supplementary material for: Genetic and Biochemical Dissection of a HisKA Domain Identifies Residues Required Exclusively for Kinase and Phosphatase Activities
Source: PLoS Genet. 2012 Nov 29;8(11):e1003084. doi: 10.1371/journal.pgen.1003084 (PMC3510030; doi:10.1371/journal.pgen.1003084)
Supplement: Table S2 — Primers used in this study. (DOCX) [file pgen.1003084.s008.docx]

**Table S2. Table of Primers used in this study**

| **Primer #** | **Primer Name** | **Primer sequence (5’ -> 3’)** |
| --- | --- | --- |
| **456** | CrdS(E372A) Forward | gcgactggcgcacgcaatcaagaacccgc |
| **457** | CrdS(E372A) Reverse | gcgggttcttgattgcgtgcgccagtcgc |
| **458** | CrdS(I373A) Forward | cgactggcgcacgaagccaagaacccgctgac |
| **459** | CrdS(I373A) Reverse | gtcagcgggttcttggcttcgtgcgccagtcg |
| **460** | CrdS(K374A) Forward | cgactggcgcacgaaatcgcgaacccgctgac |
| **461** | CrdS(K374A) Reverse | gtcagcgggttcgcgatttcgtgcgccagtcg |
| **462** | CrdS(N375A) Forward | tggcgcacgaaatcaaggccccgctgacgccc |
| **463** | CrdS(N375A) Reverse | gggcgtcagcggggccttgatttcgtgcgcca |
| **464** | CrdS(P376A) Forward | gcacgaaatcaagaacgcgctgacgcccatc |
| **465** | CrdS(P376A) Reverse | gatgggcgtcagcgcgttcttgatttcgtgc |
| **466** | CrdS(L377A) Forward | cgaaatcaagaacccggcgacgcccatccgcatg |
| **467** | CrdS(L377A) Reverse | catgcggatgggcgtcgccgggttcttgatttcg |
| **470** | CrdS(A370S) Forward | ggcgcggcgactgtcgcacgaaatcaa |
| **471** | CrdS(A370S) Reverse | ttgatttcgtgcgacagtcgccgcgcc |
| **472** | CrdS(L369A) Forward | ggaagtggcgcggcgagcggcgcacga |
| **473** | CrdS(L369A) Reverse | tcgtgcgccgctcgccgcgccacttcc |
| **474** | CrdS(R368A) Forward | ggaagtggcgcgggcactggcgcacgaa |
| **475** | CrdS(R368A) Reverse | ttcgtgcgccagtgcccgcgccacttcc |
| **503** | CrdS(Q363A) Forward | catcgccgcgtgggcggaagtggcgcgg |
| **504** | CrdS(Q363A) Reverse | ccgcgccacttccgcccacgcggcgatg |
| **505** | CrdS(V365A) Forward | gtggcaggaagcggcgcggcgac |
| **506** | CrdS(V365A) Reverse | gtcgccgcgccgcttcctgccac |
| **507** | CrdS(R367A) Forward | gcaggaagtggcggcgcgactggcgcac |
| **508** | CrdS(R367A) Reverse | gtgcgccagtcgcgccgccacttcctgc |
| **509** | CrdS(T378A) Forward | caagaacccgctggcgcccatccgcat |
| **510** | CrdS(T378A) Reverse | atgcggatgggcgccagcgggttcttg |
| **511** | CrdS(I380A) Forward | cccgctgacgcccgcccgcatgtcgctg |
| **512** | CrdS(I380A) Reverse | cagcgacatgcgggcgggcgtcagcggg |
| **513** | CrdS(M382A) Forward | gacgcccatccgcgcgtcgctggagacg |
| **514** | CrdS(M382A) Forward | cgtctccagcgacgcgcggatgggcgtc |
| **515** | CrdS(T386A) Forward | atgtcgctggaggcgctccaggccg |
| **516** | CrdS(T386A) Reverse | cggcctggagcgcctccagcgacat |
| **517** | CrdS(L387A) Forward | gtcgctggagacggcccaggccgcgcaa |
| **518** | CrdS(L387A) Reverse | ttgcgcggcctgggccgtctccagcgac |
| **519** | CrdS(N482K) Forward | cacccaggtgctggtgaagctggtgaagaac |
| **520** | CrdS(N482K) Reverse | gttcttcaccagcttcaccagcacctgggtg |
| **534** | CrdS(E372A,N375A) Forward | cgactggcgcacgcaatcaaggccccgctgacgccc |
| **535** | CrdS(E372A,N375A) Reverse | gggcgtcagcggggccttgattgcgtgcgccagtcg |
| **536** | CrdS(E372D) Forward | cgactggcgcacgatatcaagaacccgct |
| **537** | CrdS(E372D) Reverse | agcgggttcttgatatcgtgcgccagtcg |
| **538** | CrdS(I373K) Forward | cgactggcgcacgaaaagaagaacccgctgacg |
| **539** | CrdS(I373K) Reverse | cgtcagcgggttcttcttttcgtgcgccagtcg |
| **540** | CrdS(K374R) Forward | actggcgcacgaaatcaggaacccgctgac |
| **541** | CrdS(K374R) Reverse | gtcagcgggttcctgatttcgtgcgccagt |
| **542** | CrdS(K374E) Forward | tggcgcacgaaatcgagaacccgctgacg |
| **543** | CrdS(K374E) Reverse | cgtcagcgggttctcgatttcgtgcgcca |
| **544** | CrdS(N375T) Forward | ctggcgcacgaaatcaagaccccgctgacg |
| **545** | CrdS(N375T) Reverse | cgtcagcggggtcttgatttcgtgcgccag |
| **546** | CrdS(N375Q) Forward | gcgcacgaaatcaagcagccgctgacgccc |
| **547** | CrdS(N375Q) Reverse | gggcgtcagcggctgcttgatttcgtgcgc |
| **557** | CrdS(E364A) Forward | cgcgtggcaggcagtggcgcggc |
| **558** | CrdS(E364A) Reverse | gccgcgccactgcctgccacgcg |
| **559** | CrdS(A366A) Forward | tggcaggaagtgtcgcggcgactgg |
| **560** | CrdS(A366A) Reverse | ccagtcgccgcgacacttcctgcca |
| **561** | CrdS(P379A) Forward | gaacccgctgacggccatccgcatgtc |
| **562** | CrdS(P379A) Reverse | gacatgcggatggccgtcagcgggttc |
| **563** | CrdS(R381A) Forward | gctgacgcccatcgccatgtcgctggag |
| **564** | CrdS(R381A) Reverse | ctccagcgacatggcgatgggcgtcagc |
| **565** | CrdS(S383A) Forward | cccatccgcatggcgctggagacgc |
| **566** | CrdS(S383A) Reverse | gcgtctccagcgccatgcggatggg |
| **567** | CrdS(L384A) Forward | ccatccgcatgtcggcggagacgctccagg |
| **568** | CrdS(L384A) Reverse | cctggagcgtctccgccgacatgcggatgg |
| **569** | CrdS(E385A) Forward | gcatgtcgctggcgacgctccaggc |
| **570** | CrdS(E385A) Reverse | gcctggagcgtcgccagcgacatgc |
| **571** | CrdS(E372Q) Forward | ggcgactggcgcaccagatcaagaacccgct |
| **572** | CrdS(E372Q) Reverse | agcgggttcttgatctggtgcgccagtcgcc |
| **573** | CrdS(E372G) Forward | gcgactggcgcacggaatcaagaacccgc |
| **574** | CrdS(E372G) Reverse | gcgggttcttgattccgtgcgccagtcgc |
| **575** | CrdS(I373L) Forward | cgactggcgcacgaactcaagaacccgc |
| **576** | CrdS(I373L) Reverse | gcgggttcttgagttcgtgcgccagtcg |
| **577** | CrdS(I373V) Forward | cgactggcgcacgaagtcaagaacccgc |
| **578** | CrdS(I373V) Reverse | gcgggttcttgacttcgtgcgccagtcg |
| **663** | HK1190(E214A) Forward | cgctcgcgcacgcgctgaagaaccc |
| **664** | HK1190(E214A) Reverse | gggttcttcagcgcgtgcgcgagcg |
| **665** | HK1190(N217A) Forward | cgcacgagctgaaggcccccctcaccgcaa |
| **666** | HK1190(N217A) Reverse | ttgcggtgaggggggccttcagctcgtgcg |
| **667** | HK1190( E214AN217A)Forward | gctcgcgcacgcgctgaaggcccccctcaccg |
| **668** | HK1190(E214A/N217A) Reverse | cggtgaggggggccttcagcgcgtgcgcgagc |
| **669** | HK4262(E484A) Forward | ggtatcgcgcatgcgattggcacgccg |
| **670** | HK4262(E484A) Reverse | cggcgtgccaatcgcatgcgcgatacc |
| **671** | HK4262(T487A) Forward | catgagattggcgcgccgctgggcg |
| **672** | HK4262(T487A) Reverse | cgcccagcggcgcgccaatctcatg |
| **673** | HK4262(E484AT487A) Forward | tatcgcgcatgcgattggcgcgccgctgggc |
| **674** | HK4262(E484A/T487A) Reverse | gcccagcggcgcgccaatcgcatgcgcgata |
| **704** | HK853 O/E NdeI Forward | aaaacatatggttgaaaatgtgacaga |
| **705** | HK853 O/E SalI Reverse | aaaagtcgactcaattatcttgtctat |
| **706** | RR0468 O/E BamHI Forward | aaaaggatccgtgtctaaaaaagttct |
| **707** | RR0468 O/E XhoI Reverse | aaaactcgagtcattcatttaatagat |
| **708** | HK853(E261A) Forward | cgaacatctcgcacgcgctcagaacgccttt |
| **709** | HK853(E261A) Reverse | aaaggcgttctgagcgcgtgcgagatgttcg |
| **710** | HK853(T264A) Forward | acatctcgcacgagctcagagcgcctttaacg |
| **711** | HK853(T264A) Reverse | cgttaaaggcgctctgagctcgtgcgagatgt |
| **712** | HK853(E261A/T264A) Forward | gaacatctcgcacgcgctcagagcgcctttaacggcc |
| **713** | HK853(E261A/T264A) Reverse | ggccgttaaaggcgctctgagcgcgtgcgagatgttc |
