# Supplementary material for: Genetic and Biochemical Dissection of a HisKA Domain Identifies Residues Required Exclusively for Kinase and Phosphatase Activities
Source: PLoS Genet. 2012 Nov 29;8(11):e1003084. doi: 10.1371/journal.pgen.1003084 (PMC3510030; doi:10.1371/journal.pgen.1003084)
Supplement: Table S3 — List of 100 Most Diverse HisKA domains. GI numbers of 100 most diverse HisKA domains as determined by BLAST. Data were used to make the sequence logo depicted in Figure 2D. (DOCX) [file pgen.1003084.s009.docx]

**Table S3: List of 100 most sequence diverse HisKA domains**

| **#** | **GI Number and accession information for each protein** |
| --- | --- |
| **1** | GI\|157878556\|pdb\|1JOY\|A |
| **2** | GI\|108759412 |
| **3** | GI\|2623815 |
| **4** | GI\|15643616\|ref\|NP_228662.1\| |
| **5** | GI\|8953946\|gb\|AAF82192.1\|AF258464_1 |
| **6** | GI\|17229920\|ref\|NP_486468.1\| |
| **7** | GI\|16126740\|ref\|NP_421304.1\| |
| **8** | GI\|15613717\|ref\|NP_242020.1\| |
| **9** | GI\|2218094 |
| **10** | GI\|1237202 |
| **11** | GI\|14133665\|gb\|AAK54095.1\|AF362376_1 |
| **12** | GI\|15889688\|ref\|NP_355369.1\| |
| **13** | GI\|2353767 |
| **14** | GI\|13475583\|ref\|NP_107147.1\| |
| **15** | GI\|2765035 |
| **16** | GI\|15893609\|ref\|NP_346958.1\| |
| **17** | GI\|17158741\|ref\|NP_478252.1\| |
| **18** | GI\|4104609 |
| **19** | GI\|17433741\|sp\|P14376\|RCSC_ECOLI |
| **20** | GI\|15790246\|ref\|NP_280070.1\| |
| **21** | GI\|7388008\|sp\|P71380\|PHOR_HAEIN |
| **22** | GI\|15806193\|ref\|NP_294898.1\| |
| **23** | GI\|15837931\|ref\|NP_298619.1\| |
| **24** | GI\|3599372 |
| **25** | GI\|17232681\|ref\|NP_489229.1\| |
| **26** | GI\|5566398\|gb\|AAD45389.1\|AF166333_1 |
| **27** | GI\|15837455\|ref\|NP_298143.1\| |
| **28** | GI\|12229707\|sp\|Q9HWR3\|BPHY_PSEAE |
| **29** | GI\|18310494\|ref\|NP_562428.1\| |
| **30** | GI\|1352397\|sp\|P49333\|ETR1_ARATH |
| **31** | GI\|3401947 |
| **32** | GI\|17228724\|ref\|NP_485272.1\| |
| **33** | GI\|17231597\|ref\|NP_488145.1\| |
| **34** | GI\|17229733\|ref\|NP_486281.1\| |
| **35** | GI\|16804716\|ref\|NP_466201.1\| |
| **36** | GI\|18310174\|ref\|NP_562108.1\| |
| **37** | GI\|417167 |
| **38** | GI\|16127853\|ref\|NP_422417.1\| |
| **39** | GI\|8885860\|gb\|AAF80269.1\|AF155506_3 |
| **40** | GI\|14133650\|gb\|AAK54090.1\|AF362371_1 |
| **41** | GI\|15599971\|ref\|NP_253465.1\| |
| **42** | GI\|18309968\|ref\|NP_561902.1\| |
| **43** | GI\|1161055 |
| **44** | GI\|1346374 |
| **45** | GI\|15900028\|ref\|NP_344632.1\| |
| **46** | GI\|12484567\|gb\|AAG09630.1\| |
| **47** | GI\|18309217\|ref\|NP_561151.1\| |
| **48** | GI\|18311313\|ref\|NP_563247.1\| |
| **49** | GI\|17549772\|ref\|NP_523112.1\| |
| **50** | GI\|729184 |
| **51** | GI\|16126760\|ref\|NP_421324.1\| |
| **52** | GI\|17546454\|ref\|NP_519856.1\| |
| **53** | GI\|6578850\|gb\|AAF18102.1\|AF204400_2 |
| **54** | GI\|1706359 |
| **55** | GI\|138580 |
| **56** | GI\|15614576\|ref\|NP_242879.1\| |
| **57** | GI\|18307420\|emb\|CAD21033.1\| |
| **58** | GI\|15807236\|ref\|NP_295966.1\| |
| **59** | GI\|15893582\|ref\|NP_346931.1\| |
| **60** | GI\|3025461 |
| **61** | GI\|2500767\|sp\|P76339\|YEDV_ECOLI |
| **62** | GI\|16123654\|ref\|NP_406967.1\| |
| **63** | GI\|15925068\|ref\|NP_372602.1\| |
| **64** | GI\|15615987\|ref\|NP_244292.1\| |
| **65** | GI\|19554153\|ref\|NP_602155.1\| |
| **66** | GI\|14626713\|gb\|AAK71637.1\|AF388670_1 |
| **67** | GI\|15600358\|ref\|NP_253852.1\| |
| **68** | GI\|17230613\|ref\|NP_487161.1\| |
| **69** | GI\|1171792 |
| **70** | GI\|4511975\|gb\|AAD21535.1\| |
| **71** | GI\|6318167\|emb\|CAB60253.1\| |
| **72** | GI\|17228726\|ref\|NP_485274.1\| |
| **73** | GI\|13470935\|ref\|NP_102504.1\| |
| **74** | GI\|17228348\|ref\|NP_484896.1\| |
| **75** | GI\|20090134\|ref\|NP_616209.1\| |
| **76** | GI\|15597172\|ref\|NP_250666.1\| |
| **77** | GI\|6226707 |
| **78** | GI\|16127116\|ref\|NP_421680.1\| |
| **79** | GI\|15891169\|ref\|NP_356841.1\| |
| **80** | GI\|16127106\|ref\|NP_421670.1\| |
| **81** | GI\|13472175\|ref\|NP_103742.1\| |
| **82** | GI\|9857983\|gb\|AAG00949.1\|AF273679_3 |
| **83** | GI\|16262960\|ref\|NP_435753.1\| |
| **84** | GI\|16764736\|ref\|NP_460351.1\| |
| **85** | GI\|2961075 |
| **86** | GI\|15596533\|ref\|NP_250027.1\| |
| **87** | GI\|128494\|sp\|P15939\|NODV_BRAJA |
| **88** | GI\|6136818\|dbj\|BAA85819.1\| |
| **89** | GI\|17547642\|ref\|NP_521044.1\| |
| **90** | GI\|74625422\|sp\|Q9P7Q7\|MAK1_SCHPO |
| **91** | GI\|22001559\|sp\|O32193\|CSSS_BACSU |
| **92** | GI\|33112649\|sp\|P08401.2\|CREC_ECOLI |
| **93** | GI\|81669578\|sp\|O53895.1\|MPRB_MYCTU |
| **94** | GI\|54036427\|sp\|Q8DMT2\|SASA_SYNEL |
| **95** | GI\|399098\|sp\|P30844.1\|BASS_ECOLI |
| **96** | GI\|85700396\|sp\|P0C0Z0\|REGB_RHOSH |
| **97** | GI\|2499554\|sp\|Q39557\|PHY2_CERPU |
| **98** | GI\|93141316\|sp\|P39664\|SPHS_SYNP7 |
| **99** | GI\|81718236\|sp\|Q82EB2.1\|CSEC_STRAW |
| **100** | GI\|123724445\|sp\|Q2JKD9.1\|SASA_SYNJB |
