# Supplementary material for: Genetic and Biochemical Dissection of a HisKA Domain Identifies Residues Required Exclusively for Kinase and Phosphatase Activities
Source: PLoS Genet. 2012 Nov 29;8(11):e1003084. doi: 10.1371/journal.pgen.1003084 (PMC3510030; doi:10.1371/journal.pgen.1003084)
Supplement: Table S4 — List of protein sequences and Accession numbers. List of protein sequences and accession numbers of proteins used to generate Figure 2C (DOCX) [file pgen.1003084.s010.docx]

**Table S4: List of protein sequences and Accession numbers**

| **Protein Name** | **Organism** | **Amino acids shown in Figure 2C** | **GI Accession Number** |
| --- | --- | --- | --- |
| EnvZ | *Escherichia coli* | aa235-285 | 16131281 |
| NtrB | *Escherichia coli* | aa131-186 | 16131709 |
| CrdS | *Myxococcus xanthus* | aa361-423 | 108759412 |
| HK1190 | *Myxococcus xanthus* | aa205-265 | 108760457 |
| HK4262 | *Myxococcus xanthus* | aa475-531 | 108759953 |
| HK853 | *Thermotoga maritima* | aa252-310 | 15643616 |
