# Supplementary material for: Genetic and Biochemical Dissection of a HisKA Domain Identifies Residues Required Exclusively for Kinase and Phosphatase Activities
Source: PLoS Genet. 2012 Nov 29;8(11):e1003084. doi: 10.1371/journal.pgen.1003084 (PMC3510030; doi:10.1371/journal.pgen.1003084)
Supplement: Text S1 — Supplemental Materials and Methods: Purification protocols for Thermotoga maritima proteins and for synthesis of radiolabeled acetyl-phosphate. (DOCX) [file pgen.1003084.s011.docx]

**Supplemental Methods**

**Generation of Radiolabeled Acetyl Phosphate.** Response regulators were labeled *in vitro* using the high energy phosphor-donor acetyl phosphate (AcP). [^32^P]AcP was synthesized, using acetate kinases (AcK) purified from *E. coli* (Sigma) during a one hour room-temperature incubation (0.25 Units of AcK, 5 μL [γ-^32^P]ATP, 12.5mM Tris-HCl pH8.0, 30 mM Potassium Acetate, 5 mM MgCl_2_). AcK was removed by passing over a Amicon Ultra 0.5 mL 10k spin column (Millipore) and collecting the flow through containing [^32^P]AcP. Labeling occurred by incubating RR wit [^32^P]AcP at room temperature (5 μM RR with equal volume AcP). Unincorporated [^32^P]AcP was removed by running sample over a Zeba 7K MWCO desalting column equilibrated in 1X kinase buffer.

**Expression and purification of *T. maritima* proteins.**

A soluble form of *Thermotoga maritima* HK853 (expressing amino acids 232-489) was cloned into the IPTG (isopropyl-β-ᴅ-thiogalactopyranoside) inducible vector pET28a. The full-length RR468 protein was also cloned into pET28a for purification (Novagen). All point mutations were generated using site-directed mutagenesis and verified by sequencing. All proteins were expressed using the *E.* coli strain BL21(DE3). For overexpression, 1-liter of Terrific Broth was inoculated with 25mL of overnight culture and grown at 37°C with shaking until the OD_600_ (optical density at 600 nm) reached 0.4 to 0.6. Cultures were then induced by addition of 0.5 mM IPTG and grown overnight at 20°C, pelleted by centrifugation then stored at -20°C until purification.

All *T. maritima* proteins were purified using a batch purification method. Frozen cell pellets were resuspended in 10 mL Lysis buffer (25 mM Tris pH7.6, 125 mM NaCl, mini-EDTA Free protease inhibitor-Roche) and lysed using CelLtyic (Sigma). Lysates were clarified by centrifugation at 50,000 x *g*. Samples were then incubated at 75°C for 1 hour, and followed by additional centrifugation at 50,000 x *g* to remove insoluble debris. To the clarified lysate, 2 mL His-select Cobalt affinity gel (Sigma) was added which was equilibrated with Lysis buffer. Samples were incubated at 4°C for 1 hour with mild shaking. The resin was pelleted by a one minute spin at 100 x *g* and washed 3 X with 10 mL Lysis buffer. Protein was eluted using Elution buffer (25 mM Tris pH7.6, 125 mM NaCl, 500mM Immidazole) and dialyzed overnight against one liter of Dialysis buffer (25 mM Tris pH7.6, 125 mM NaCl, 1 mM DTT, 1% Triton X100, 50% glycerol). Samples were stored at – 20°C until use. Sample purity was assessed using Coomassie stained SDS-PAGE gels. Protein concentrations were determined using Bradford Reagent (Biorad).

***In vitro*** **autokinase and phosphatase assays.**  Autokinase reactions we performed by incubating 5 μM HK in 1 X kinase buffer (25mM Tris pH 7.6, 50mM KCl, 1mM of CaCl_2_ MgCl_2_ and MnCl_2_ , 1mM β-mercaptoenthanol) and ATP mix (250 μM ATP, 0.3 μM [γ-^32^P]ATP). Reactions were stopped by addition to an equal volume of 2X SDS-Loading Buffer. Samples were resolved by electrophoresis on 10% SDS polyacrylamide gels. The dye front, containing unincorporated ATP, was removed. Gels were exposed for 1-3 hours on a phosphor screen and then visualized using a Typhoon Imager. ImageQuant was used to determine pixel density.

Phosphatase experiments were carried out using RR468~P phosphorylated with radiolabeled acetyl phosphate ([^32^P]AcP). After RR468~^32^P was generated, it was incubated with equal molar amounts (5 μM each) of HK853 and incubated for a period of 1 hour before samples were separated using SDS-PAGE with subsequent exposure and visualization using phosphor screens.
